# Molecular regulation of NLRP3 inflammasome activation during parasitic infection

**DOI:** 10.1042/BSR20231918

**Published:** 2024-05-15

**Authors:** Rasha Alonaizan

**Affiliations:** Faculty of Science, King Saud University, P.O. Box 2455, Riyadh 11451, Saudi Arabia

**Keywords:** Helminths, NLRP3 Inflammasomes, Plasmodium, T. gondii, Trypanosome cruzi

## Abstract

Parasitic diseases are a serious global health concern, causing many common and severe infections, including Chagas disease, leishmaniasis, and schistosomiasis. The NLRP3 inflammasome belongs to the NLR (nucleotide-binding domain leucine-rich-repeat-containing proteins) family, which are cytosolic proteins playing key roles in the detection of pathogens. NLRP3 inflammasomes are activated in immune responses to *Plasmodium, Leishmania, Toxoplasma gondii*, *Entamoeba histolytica, Trypanosoma cruzi*, and other parasites. The role of NLRP3 is not fully understood, but it is a crucial component of the innate immune response to parasitic infections and its functions as a sensor triggering the inflammatory response to the invasive parasites. However, while this response can limit the parasites’ growth, it can also result in potentially catastrophic host pathology. This makes it essential to understand how NLRP3 interacts with parasites to initiate the inflammatory response. *Plasmodium* hemozoin, *Leishmania* glycoconjugate lipophosphoglycan (LPG) and *E. histolytica* Gal/GalNAc lectin can stimulate NLRP3 activation, while the dense granule protein 9 (GRA9) of *T. gondii* has been shown to suppress it. Several other parasitic products also have diverse effects on NLRP3 activation. Understanding the mechanism of NLRP3 interaction with these products will help to develop advanced therapeutic approaches to treat parasitic diseases. This review summarizes current knowledge of the NLRP3 inflammasome’s action on the immune response to parasitic infections and aims to determine the mechanisms through which parasitic molecules either activate or inhibit its action.

## Introduction

Inflammasomes are intracellular multimeric complexes playing key roles in innate immunity against numerous pathogens and physiological stimuli, their action is important in regulating inflammatory response. Since excessive inflammation can be harmful to cells and tissues, whereas inadequate inflammation response can be beneficial for pathogens. Innate immunity response mostly involves the detection of pathogens or danger associated molecular patterns (PAMPs and DAMPs, respectively) by pattern recognition receptors (PRRs) such as Toll-like receptors TLRs, NOD-like receptors (NLRs), C-type lectin receptors (CLRs), and RIG-I-like receptors (RLRs) [1,2]. This will incuse a signaling cascade resulting in triggering inflammation response in attempting for the agent clearance. Contrasting with the other known PRRs, some members of the NLR family are unique in their capability to form an inflammasome complex to activate caspase-1, an enzyme that cleaves proinflammatory interleukin-1β (IL-1β) and interleukin-18 (IL-18) leading to inflammation and pyroptosis a form of cell death of the infected cell [3].

Inflammasomes are parts of the innate response that contribute to the inflammatory response by stimulating the caspase-1 inflammatory pathway, resulting in the maturation of interleukin-1β (IL-1β) and interleukin-18 (IL-18), and pyroptotic cell death [4]. The inflammasome is a signaling platform with three components (sensor, adaptor, and effector) that begin to form when endogenous and/or external threats are detected. Resulting in successive oligomerization of pro-caspase-1 to effecter caspase-1. Inflammasomes are formed from five-member proteins, the nucleotide-binding oligomerization domain (NOD), leucine-rich repeat (LRR)-containing proteins, the NLR family members NLRP1, NLRP3, and NLRC4, the absent-in-melanoma 2 (AIM2), and pyrin (a bipartite adaptor protein) [5,6].

The NLRP3 inflammasome is critical for host immune defences against several types of infections, including bacterial, fungal, and viral [7–10]. It is mostly expressed as a result of inflammatory stimulation in antigen-presenting cells (APCs) such as macrophages, dendritic cells (DC), neutrophils, and monocytes [11]. Interestingly, NLRP3 activation has been associated with the pathogenesis of certain inflammatory conditions, including cryopyrin-associated periodic syndromes (CAPS), Alzheimer’s disease, diabetes, gout, autoinflammatory disease, and atherosclerosis [12,13]. NLRP3 is a 118 kDa cytosolic protein expressed by a diversity of cells like lymphocytes, osteoblasts and neurons in addition to APCs. Its structure includes a multilateral protein complex containing an amino-terminal pyrin domain (PYD), which recruits proteins for inflammasome complex formation [14,15]. A central nucleotide-binding and oligomerization domain (NOD, NACHT domain), and a C-terminal leucine-rich repeat (LRR) domain [16]. The pyrin domain of NLRP3 interacts with the pyrin domain of ASC to trigger inflammasome formation [17]. Similar to other inflammasomes, the NLRP3 inflammasome complex contains a sensor (NLRP3 protein), an adaptor (apoptosis-associated speck-like protein, ASC), and an effector (caspase-1) [18,19]. NLRP3 is unable to bind to stimuli directly, and instead senses the frequent cellular signals triggered by their presence. Current models of the classical or canonical NLRP3 activation divide into two signaling steps, priming (signal 1) and activation (signal 2), in addition to non-canonical activation pathway [20].

### The NLRP3 inflammasome activation mechanism

#### The priming signal (signal 1)

The priming signal (signal 1) is the first step required for NLRP3 inflammasome activation, that is responsible for the transcriptional up-regulation of NLRP3 and pro-interleukin (IL)-1β and pro-IL-18 [21]. It occurs when a cell exposed to priming stimuli like LPS or necrosis factor (TNF) and IL-1β through TLRs, tumor necrosis factor receptor (TNFRs), NOD2, IL-1R respectively. The detection of these inflammatory stimuli causes the activation of proteins and nuclear factors such as myeloid differentiation primary response protein (MyD88), nuclear factor kappa-light-chain-enhancer of activated B cells (NF-κB) to increase NLRP3 and IL-1β transcription, leading to up-regulation of NLRP3 protein and pro-IL-1β [22]. However, when these component molecules translocate from nucleus to cytoplasm, they are in inactive forms and require a second signal to be activated [23].

#### NLRP3 inflammasome activation (signal 2)

The second signal facilitates the oligomerization of the inactive inflammasome complex (NLRP3, ASC, and pro caspase-1), leading to the maturation, and up-regulation of the pro-IL-1β and pro-IL-18 [24,25]. Conflicting to other PRRs, NLRP3 can be activated by abundance of stimuli such as uric acid crystals, silica, asbestos, extracellular ATP, and toxins, plus to viral, bacterial, fungal, and protozoan molecules [26,27]. In addition, the second signal can be induced by several molecular events such as ionic flux, mitochondrial dysfunction, the production of reactive oxygen species (ROS), and lysosomal damage [28]. Although it is uncertain how NLRP3 is able to identify such different signals, it was proposed that NLRP3 senses a common cellular incident resulted by all stimuli rather than direct binding to them. Following the signal 2, the adaptor protein ASC and inactive pro-caspase-1 join together, then subsequently cleaving pro-caspase-1 into active caspase-1, which in turn cleaves pro-IL-1β and pro-IL-18 into their active form, plus activates the membrane pore-forming gasdermin D (GSDMD). GSDMDs N-terminal domain (GSDMD-NT) protein cleaves and oligomerizes to form pores in the cell membrane resulting in pyroptosis and the release of intracellular components, including inflammatory cytokines IL-1 β and IL-18 [29].

#### NLRP3 inflammasome activation via the non-canonical pathway

NLRP3 inflammasome can be activated indirectly via a non-canonical pathway with the enrolment of caspase-11 in mice or the human analogs caspase-4/5. This non-canonical NLRP3 inflammasome pathway involves the direct senses and binding between these caspases and cytoplasmic LPS through TLR4, that will eventually result in oligomerization and activation of NLRP3 inflammasome followed by the secretion of IL-1β/IL-18 and pyroptosis [30,31].

Several studies have found that the NLRP3 inflammasome responds are key in controlling bacterial pathogens [32]. Recently further studies have suggested that the NLRP3 inflammasome also plays an important role in the host’s response to protozoan infection [33] This review focuses on current advances in research on NLRP3 inflammasome activation and its inflammatory response during different parasitic infections. In addition, it examines the immune evasion mechanisms of parasitic molecules that target the NLRP3 inflammasome response. We also outline novel approaches targeting NLRP3 signaling that could be developed as therapeutic alternatives to current anticancer treatment.

### NLRP3 actions during parasitic infection

#### Plasmodium

Malaria is one of the most common infectious diseases caused by *Plasmodium* species and leads to worldwide human morbidity and mortality [34]. According to the World Health Organization (WHO), in 2020, an estimated 241 million new cases of malaria were recorded worldwide, resulting in half a million deaths [35]. Malaria infections can be asymptomatic, have only mild symptoms, or be fatal, depending on factors such as parasite virulence and host genetics [36]. Malaria symptoms are characterized by periodic paroxysms, severe anemia and headaches, and can lead to metabolic, renal, and cerebral complications that can be fatal in untreated individuals [20].

*Plasmodium* is a eukaryotic organism capable of morphological alterations during its complex life cycle which includes both sexual and asexual stages within two different hosts [34]. The *Plasmodium* species that infect humans are *P. falciparum, P. vivax, P. malariae, P. ovale, and P. knowlesi*, with *P. falciparum* being the most dangerous to humans [37,38]. *Plasmodium* is transmitted by a female *Anopheles* mosquito when it feeds on the host’s blood. Once in the host, the parasite enters the blood stage of its development, which in humans is the stage that causes the pathology of malaria [23,39]. A strong immune response is therefore necessary to control the early infection and reduce disease severity [40].

The parasite produces several immunomodulatory molecules, such as glycosylphosphatidylinositols anchor (GPIs), hemozoin (Hz), and immunostimulatory DNA, that trigger strong innate immune mechanisms, including the production of phagocytic cells, NK cells and the expression of inflammasome-related genes such as MyD88, caspase-1, ASC, P2X7R, and NLRP3 [41,42]. The innate immune response to malaria infection is crucial to the development of the adaptive immunity needed to regulate parasite pathogenesis [43]. This adaptive immunity includes promoting Th1 responses to produce proinflammatory cytokines, such as IL-1β, IL-18, IL-12, tumor necrosis factor (TNF-α), and interferon (IFN)-γ to effectively clear the infection. However, under some conditions, the immune system may fail, resulting in a proinflammatory storm of cytokines such as IL-1β, IL-18, TNF-α, and IFN-γ that associated with increased disease severity and poorer clinical outcomes [44,45]. However, many details of the immune response against intracellular parasites, including Malaria, are not fully understood.

**Figure 1:**
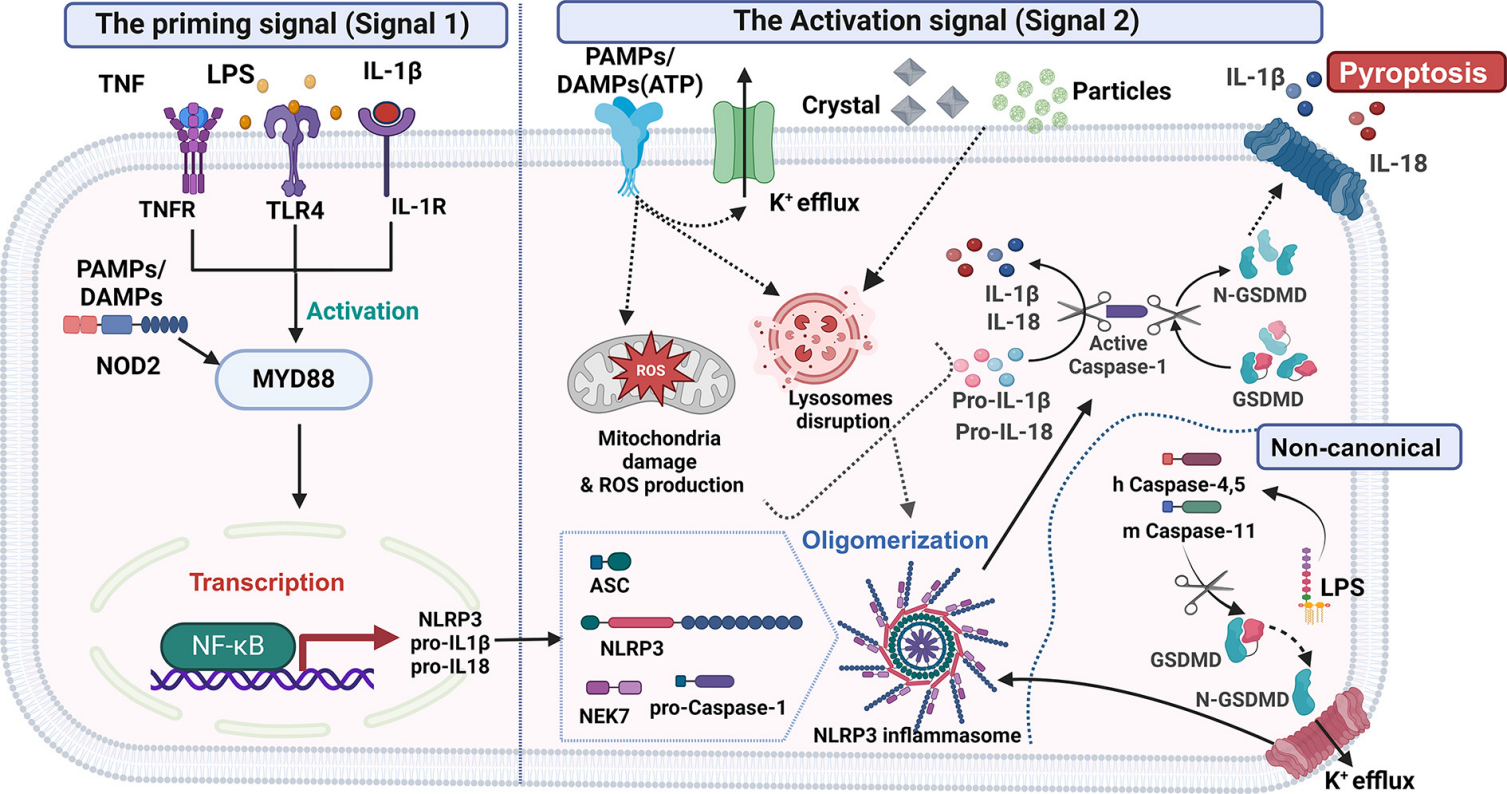
Mechanisms of activation of NLRP3 inflammasomes **The priming signal (Signal 1)** is the first phase in inducing the transcriptional up-regulation of NLRP3, pro-interleukin β (IL-1β) and pro-IL-18. It begins when pathogen‐associated molecular patterns (PAMPs) and damage‐associated molecular patterns (DAMPs), such as LPS, necrosis factor (TNF) and IL-1β bind to Toll-like receptors (TLRs), tumor necrosis factor receptor (TNFRs), nucleotide binding oligomerization domain containing 2 (NOD2) and interleukin-1 receptor (IL-1R), respectively. This results in the transcriptional up-regulation of NLRP3, IL1 β, and IL18 via activation of myeloid differentiation primary response protein (MyD88) proteins and transcription factors nuclear factor kappa-light-chain-enhancer (NF-κB). **The activation signal (Signal 2)** is the second signal that triggered by PAMPs or DAMPs, such as adenosine triphosphate (ATP) and crystals which stimulate diverse signaling events including ROS, lysosomal damage and K+ efflux, resulting in oligomerization, and activation of NLRP3 inflammasome complex. The activation of NLRP3 inflammasome leads to two events: (i) When the adaptor protein ASC and inactive pro-caspase-1 couple together, afterwards cleaving pro-caspase-1 into active caspase-1, which sequentially cleaves the pro-IL-1β and pro-IL-18 into their bioactive forms preceding their release. (ii) active caspase-1 also cleaves Gasdermin D into N- GSDMD, therefore pyroptosis induction and IL-1β and IL-18 production. **Non-canonical NLRP3 inflammasome activation** is prompted by the cytosolic LPS detecting by human caspase 4/5 or mouse caspase 11, followed by cleaving and formation of GSDMD membrane pores, leading to potassium efflux, which eventually triggers NLRP3 inflammasomes activation. The activated NLRP3 cleaves the GSDMD to form additional membrane pores and induce the active form of caspase-1, pro-IL-1β and pro-IL-18, resulting in pyroptotic cell death.

NLRP3 inflammasome is a critical part of innate immune response and its activation is an important antimalarial mechanism. During infection NLRP3 inflammasome can be activated by erythrocyte *Plasmodium* molecules [46,47]. However, it is still unknown whether the inflammasome activation has a beneficial or harmful impact on host immunity and mortality during lethal malaria infection. Therefore, several studies have been conducted to understand the interaction of NLRP3 inflammasome with *Plasmodium* during infection. The parasite feeds on the hemoglobin of red blood cells and generates a metabolic waste called hemozoin (Hz). A study using IL-1β deficient mice showed that Hz can induce IL-1β production via NLRP3 activation. The underlying signaling mechanism by which Hz triggers NLRP3 pathway activation and IL-1β production involves the Src kinase Lyn and the tyrosine kinase Syk (Table 1) [48] Moreover, Hz-dependent activation of NLRP3 can be enhanced by uric acid released during malaria infection and suppressed by allopurinol (an inhibitor of uric acid synthesis) [49] (Figure 1). Velagapudi et al. found that incubating BV-2 microglia with HZ increases NLRP3 expression and caspase-1 activity [50]. Accordingly, these findings indicate that the ability of plasmodium product HZ to induce inflammasome action.

**Table 1:** Summary of parasitic molecules and their actions to NLRP3

Parasite Name	Parasite molecules	Action on NLRP3	By means	Result	Ref.
*Plasmodium*	Hemozoin (Hz)	Activation	Src kinase Lyn and the tyrosine kinase Syk	IL-1β production	[36]
			?	Negatively influences conventional CD8a+ type 1 dendritic cell (cDC1) abundance, phagocytosis	[42]
	Uric acid	Enhance	?	IL-1β production	[37]
	Hz coated with plasmodial genomic DNA (gDNA) or CpG oligonucleotides	Activation	TLR9		[43]
*Leishmania*	Parasite membrane glycoconjugatelipophosphoglycan (LPG)	Activation	CASP11 activation in macrophages and *in vivo*		[51]
	GP63 factor	Suppression	?	Reduction of IL-1β production	[52]
	RNA virus (LRV) virulence factor		TLR3 and TRIF	Leading to Autophagy-related 5 (ATG5) expressions mediating NLRP3 breakdown	[35]
*T. gondii*	The soluble total Ag (STAg) derived from *T. gondii* strain RH	Activation	?	Increasing IL-1β secretion *in vitro*	[53]
	Dense granule proteins 15 (GRA15)		?	L-1β and IFN-γ production	[54]
	*T. gondii* secretory protein, rhoptry protein 7 (ROP7)	Hyperactivation	IL-1β/NF-κB/ NLRP3 pathway	Up-regulation NF-κB expression	[50]
	Dense granule proteins 9 (GRA9)	Suppression	?	Anti-inflammation response	[55]
*E. histolytica*	Gal/GalNAc lectin	Activation	Activate NF-κB and MAP kinase-signaling pathways	Pro-IL-1β	[56]
	EhCP-A5 RGD binding with macrophages α5β1 integrin		Src family kinase phosphorylation and opening of Panx1	Release of ATP	[57]
	Peroxiredoxins (Prx)		Binding with TLR4 receptor and P2X7		[58]
	Prostaglandin E2 PGE2	Suppression	Coupling E-prostanoid 4 (EP4)	Turned off ATPase affecting self-oligomerization of NLRP3	[59]
*T. cruzi*	*T. cruzi* antigen (TcAg)	Suppression	?		[60]
*Schistosomal*	Soluble schistosomal egg antigens (SEA)	Activation	SEA protein functions as a second signal	Resulting in IL-1β production in dendritic cells	[61]
*Fasciola hepatia*	FhCL3 helminth-derived molecules of Fasciola hepatica	Activation	?	Promoting adaptive immune response,	[62]
	Fasciola hepatica products like FhHDM-1 (cathelicidin-like peptide)	Suppression	?	Reduction in IL-1β secretion by macrophages	[63]

NLRP3 activation may also induce neuroinflammation during cerebral malaria (CM), a type of malaria with high mortality and affecting approximately 3 million individuals each year [52]. High concentrations of proinflammatory cytokines and chemokines, such as TNF-α, IL-6, IL1β, IFN-γ, and CXCL10, are often correlated with the progression of CM [64]. One study found that decreasing NLRP3 activation by injecting mice with IL-33 cytokines in combination with antimalarial drugs, significantly reduced the progression of CM. Consistent with this, inhibiting the NLRP3 inflammasome directly by MCC950 phenocopied inhibitor, promotes the protective role of IL33 towards CM [65]. This suggests that the level of NLRP3 activation during *Plasmodium* infection influences CM progression, and that targeting the NLRP3 inflammasome to reduce its activation could be an excellent pharmacological strategy for treating CM.

**Figure 2:**
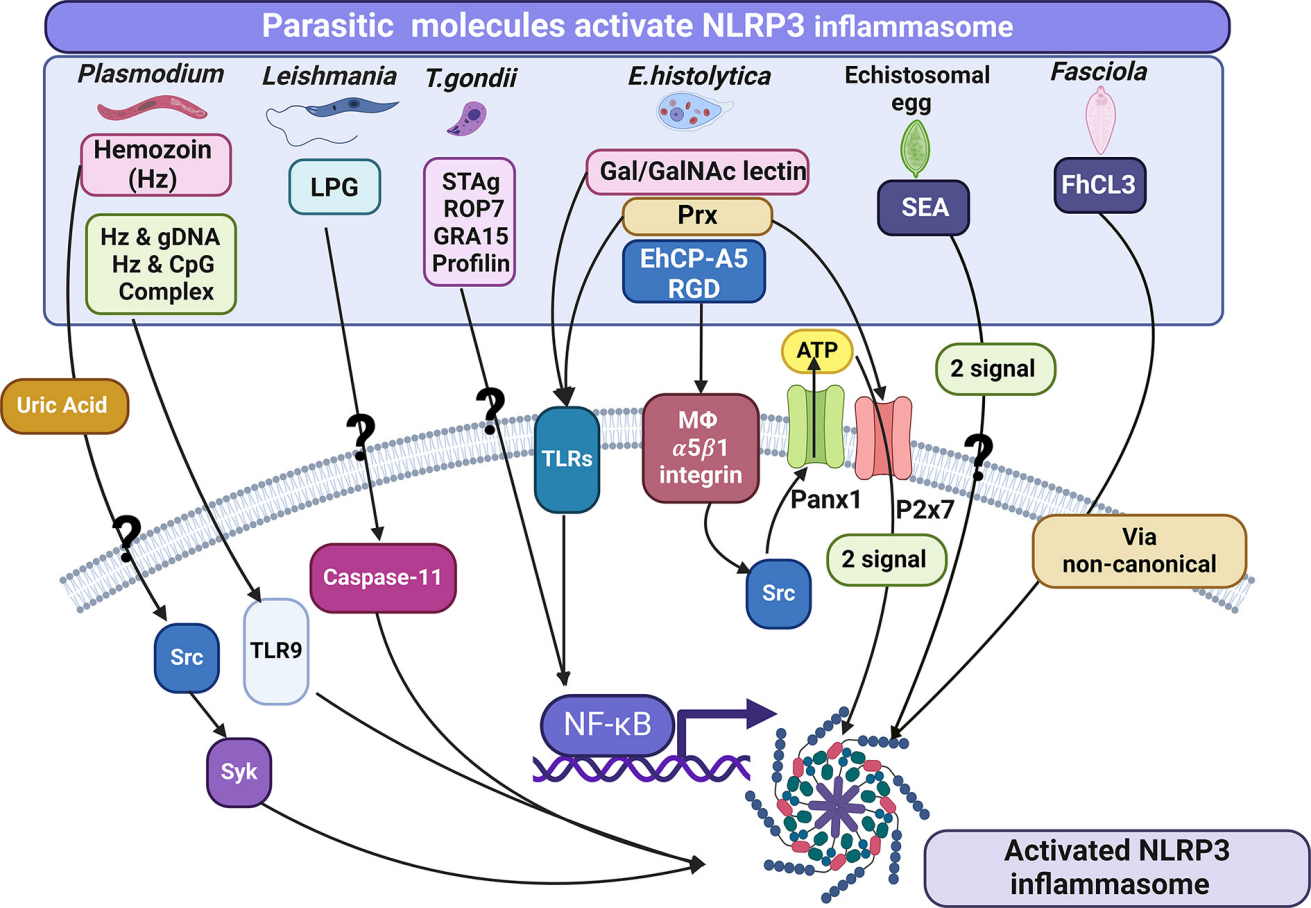
Schematic representation of the mechanisms NLRP3 activation by parasitic molecules *Plasmodium* hemozoin (Hz) is able to stimulate NLRP3 activation via the Src kinase Lyn and the tyrosine kinase Syk. Also, Hz-NLRP3 activation pathway be boosted by the uric acid produced during malaria infection. Hz coated with plasmodial genomic DNA (gDNA), or CpG-oligonucleotides initiate TLR9 translocation leading to NLRP3 activation. While Leishmania membrane glycoconjugate lipophosphoglycan (LPG) initiates the NLRP3 activation through caspase-11 pathway. *T. gondii* induces NLRP3 activation by the soluble total Ag (STAg) rhoptry protein 7 (ROP7), Profilin from *T. gondii* (TgP) and effector proteins GRA15 causing significant up-regulation in NF-κB expression and consequently inflammasome activation via the IL-1β/NF-κB/NLRP3 pathway. Galactose/ N-acetylgalactosamine (Gal/GalNac) lectin of *E. histolytica* promotes NF-κB and MAP kinase-signaling pathways resulting in NLRP3 inflammasome components and pro-IL-1β transcription. *E. histolytica* Peroxiredoxins (Prx) also functions as a key domain that causes NLRP3 activation pathway via the interaction with TLR4 receptor and P2X7 receptor. Also, Gal/GalNAc lectin supports the formation of the intracellular junction between EhCP-A5 RGD domain and α5β1 integrin resulting in activation of Src family kinase phosphorylation and pannexin-1 (Panx1) channel to enable ATP release. This free ATP then signals back via P2X7 receptors for promoting the second signal for NLRP3 inflammasome formation. In addition, Soluble schistosomal egg antigens (SEA) as a second signal can induce the activation of NLRP3 inflammasome and IL-1β. The FhCL3 helminth-derived molecules of Fasciola hepatica also can promote NLRP3 activation and IL-1β and IL-18 production in non-canonical inflammasome dependent manner.

It has been mentioned that malarial pigment Hz can active the NLRP3 inflammasome, however, this activation has negative influence in conventional CD8α+ type 1 dendritic cell (cDC1) abundance, phagocytosis and T-cell activation *in vivo* [66]. Eventually, this will advantage the parasite by reducing the effectiveness of the anti-*Plasmodium* humoral response. Hz has previously been found to carry plasmodial DNA into a subcellular compartment reachable by Toll-like receptor 9 (TLR9), resulting in inflammatory signals. An *in vitro* study applying synthetic Hz coated with plasmodial genomic DNA (gDNA), or CpG-oligonucleotides, found that DNA-complexed Hz prompted TLR9 translocation resulting in activation of the NLRP3 and AIM2 inflammasomes. These findings suggest that Hz and DNA collaborate to induce systemic inflammation during malaria [67] (Figure 2).

Many studies aim to comprehend the effects of NLRP3 deficiency during malaria. For instants, a study in NLRP3-deficient mice infected with lethal *Plasmodium yoelii YM*, found increased IFN-I cytokine production and a high survival rate in parallel with reduced IL-1β production. Other findings show that NLRP3 and IL-1β knockout mice do not experience increased body temperature during the acute phase of *P. chabaudi Adami* infection, and only exhibit mild symptoms [48]. Mice deficient in inflammasome sensors AIM2, NLRP3, or adaptor caspase-1, and infected with *Plasmodium yoelii YM*, show increased production of IFN-I cytokines and IL-1β production, and increases IFN-I production. Since inflammasome activation involves the induction of IL-1β-mediated MyD88-TRAF3-IRF3 signaling and up-regulation of suppressor of cytokine signalling 1 (SOCS1). A study found that inhibition of MyD88-IRF7-mediated-IFN-I signaling by SOCS1 reduces the cytokine production in plasmacytoid dendritic cells. In addition, the lack of inflammasome components decreases SOCS1 stimulation, causing inhibition of MyD88-IRF7-dependent-IFN-I signaling, resulting in increased IFN-α/β secretion and host survival. These effects indicate some of the negative aspects of inflammasome activation in the regulation of IFN-I pathways [68]. However, IFN-I pathways show conflicting roles during *Plasmodium* infections, due to the organism’s complex life cycle (Figure 2).

NLRP3 has been targeted in many malaria vaccines, such as QS-21, a soluble saponin adjuvant that induces IL-1β/IL-18 production and promotes Th1 responses in macrophages and dendritic cells [69]. Developing vaccination candidates against malaria that target the NLRP3 pathway, may lead to better infection control. However, examining the role of NLRP3 activation in vaccine development for malaria is beyond the scope of this review.

It may conclude that plasmodium molecules such as Hz and (gDNA), and CpG-oligonucleotides have an immunostimulatory effect in NLRP3 inflammasome. While there is no confirmed direct interaction between GPI anchors and NLRP3, a study indicated that GPIs can activate TLRs to produce proinflammatory cytokines such as IL-1β [70]. Moreover, activation of NLRP3 *Plasmodium* infections can worsen CM progression because of its suppressant action on IL-33 production. In addition, activation of NLRP3 can benefit the parasite by reducing both T cell activation and the humoral response, resulting in worsening infection and poorer prognoses. In contrast, lower NLRP3 activation increases the production of IFN-I cytokines and reduces disease symptoms. These results imply that the inhibition of inflammasome activation could be a valuable target in the development of effective malaria treatments.

#### Leishmania

*Leishmania* is an intracellular parasite that can cause leishmaniasis, a tropical and subtropical infectious disease. *Leishmania* is transmitted to humans by the bite of sandflies such as *Phlebotomus* and *Lutzomyia* [71]. Leishmaniasis has been characterized by WHO as one of the seven most important tropical diseases and is prevalent in North East Africa, Southern Europe, the Middle East, South eastern Mexico, and Central and South America. It is a complex disease, with significant clinical and epidemiological diversity and gives rise to a broad spectrum of symptoms and in some cases can lead to death [72]. Worldwide, 1.5 to 2 million new cases occur each year, resulting in 70,000 deaths.

*Leishmania* progresses through two main developmental stages each with its own morphology: promastigotes and amastigotes. Promastigotes are able to move within the gut of the sand fly, while amastigotes live intracellularly in mammalian cells such as macrophages. More than 20 different *Leishmania* species are known to cause disease in humans like *L. major, L. mexicana, L. amazonensis, and L. brasilliensis* are the cause of cutaneous infections of the skin. However, the most severe and sometimes fatal disease is caused by *L. donovani and L. infantum*. These species infect the host systematically, resulting in visceral leishmaniasis which accounts for a total of 70,000 deaths [73]. Clinical manifestations are influenced by the species of *Leishmania* and the immune response of the host, and range from localized cutaneous infections to the potentially lethal visceral form [74].

After transmission of Leishmania parasites by sandflies, clinical manifestation of the infection requires mechanisms that allow the parasites to proliferate in the mammalian host and attack, resulting in initiating the innate and adaptive antileishmanial defence. *Leishmania* parasites’ ability to challenge the host’s immune response and eventually establish a chronic infection, makes the disease extremely difficult to treat. The rapid clearing of pathogens, and further shaping of the adaptive immune response, is vital for controlling infection and improving disease outcomes [75]. Both innate and adaptive immunity are therefore essential for the host’s defence against *Leishmania*. The innate response is initiated by a complex interaction between parasitic molecules, such as lipophosphoglycan (LPG), glycoprotein-63 (GP63), and glycosylphosphatidylinositol (GPI), and the receptors of the antigen-presenting cells (APCs) [51]. This interaction represents a type I immune response, and involves the production of IL-12 followed by IFN-γ-secreting. This leads to the initiation of the macrophages’ microbicidal mechanisms [76]. Adaptive immunity is essential for improving disease outcomes and to fully eliminate the infection and create long-lasting response memories against re-infection by *Leishmania* [36]. Several proinflammatory cytokines are secreted during the adaptive phase, such as TNF-α, IFN-γ, IL-1β, IL-12, and IL-18 which together form an inflammatory response regulating parasite growth and infection outcome [77].

Recent advances in research have indicated crucial role for NLRP3 inflammasomes during Leishmaniasis.NLRP3 inflammasomes exert strong control over IL-1β and IL-18 production, and these cytokines are considered key mediators during *Leishmania* infections both *in vitro* and *in vivo* [78,79]. One study infected mouse macrophage with different *Leishmania* species, such as *L. amazonensis, L. braziliensis*, and *L. Mexicana and* found induction of caspase-1 activation and IL-1β production was dependent on the NLRP3 inflammasome. Furthermore, NLRP3 knockout mice were found to be extremely susceptible to *L. amazonensis* infection in comparison with WT control mice, indicating the protective role of NLRP3 inflammasome activation. This protective role involves IL-1β production, and therefore NO secretion, which contributes to the *Leishmania* killing mechanism [80]. In contrast, infection of C57BL/6 mice with the *L. major Seidman strain* (*LmSd*) (isolated from a patient with chronic lesions), results in unhealed lesions, uncontrolled parasite growth and full destruction of the ear dermis. This is accompanied by IL-1β production within dermal cells and remarkable neutrophil recruitment to the infected skin. Similarly, the severity of lesions in tegumentary leishmaniasis (TL) patients has been associated with increased expression of AIM2, which is part of the NLRP3 inflammasome [81,82]. However, mice deficient in NLRP3, ASC, and caspase-1/11, or lacking IL-1β or IL-1 receptors, have better lesions repair and parasitic elimination, due to the absence of IL-1β - which affects neutrophils’ local enrolment and therefore suppresses inflammation [82]. The production of IL-1β dependent on NLRP3 inflammasome activation may be limits neutrophil recruitment, and causes non-healing forms of cutaneous leishmaniasis in commonly resistant mice. In contrast, a study found that infecting susceptible BALB/c mice with *L. major* induced severe footpad swelling and parasite burden, whereas NLRP3−/− BALB/c mice showed considerably reduced footpad swelling and the parasite burden. This suggests NLRP3 activation has a negative impact on BALB/c mice during infections with *L. major*. The authors propose that IL-18 might promote *L. major* survival by suppressing Th1 cell responses [83].

In another study aiming to understand the role of the NLRP3 inflammasome in Th1/Th2 responses during leishmaniasis, knockout BALB/c mice for NLRP3, ASC, or caspase-1, displayed deficient IL-1β and IL-18 production and were resistant to cutaneous *L. major* infection. This study also indictes that the production of IL-18 enhances disease susceptibility in BALB/c mice by stimulating anti-inflammatory cytokine production. Neutralization of IL-18 in these animals lowered the *L. major* burden and reduced footpad swelling [84]. These studies all suggest that IL-18 neutralization could be a potential pharmacological approach in the treatment of leishmaniasis patients.

Several studies have been conducted to increase our understanding of the underlying mechanisms of NLRP3 activation during leishmaniasis. For instant, inflammasome activation during the onset stages of *L. amazonensis* infection in macrophages, seems to require ROS production through the NADPH oxidase mechanism, and the engagement of Dectin-1 and a C-type lectin receptor via spleen tyrosine kinase (Syk) signals. Therefore, inflammasome activation in response to *L. amazonensis* is decreased by the deficiency of NADPH oxidase, Syk, focal adhesion kinase, and proline-rich tyrosine kinase 2, as well as by the absence of Dectin-1. Further experiments confirmed this using Dectin-1 knockout mice, where Dectin-1 inflammasome activation was found to be important in controlling the parasite burden in macrophages, and improving resistance to *L. amazonensis* infection *in vivo* [85]. An alternative pathway has been suggested to participate in the NLRP3 inflammasome activation that helps control *L. amazonensis* infection. This pathway is facilitated by the P2X7 receptor and LTB4 and depends on the production of IL-1β via non-canonical NLRP3 inflammasome activation [86]. This is supported by the finding that inflammasome genes like IL-1β, NLRP3, and P2RX7, are up-regulated in localized cutaneous leishmaniasis (LCL) patients [87]. Furthermore, Carvalho et al. found that the parasite membrane glycoconjugate lipophosphoglycan (LPG) triggers the NLRP3 inflammasome pathway via caspase-11 activation in macrophages and *in vivo* [88]. These studies propose possible pathways for the activation of the NLRP3 inflammasome during *Leishmania* infection and improve our understanding of the immunological role of NLRP3 activation in the host's immune response. Understanding these mechanisms is important for the development of new therapeutic strategies to limit leishmaniasis progression (Figure 2).

**Figure 3:**
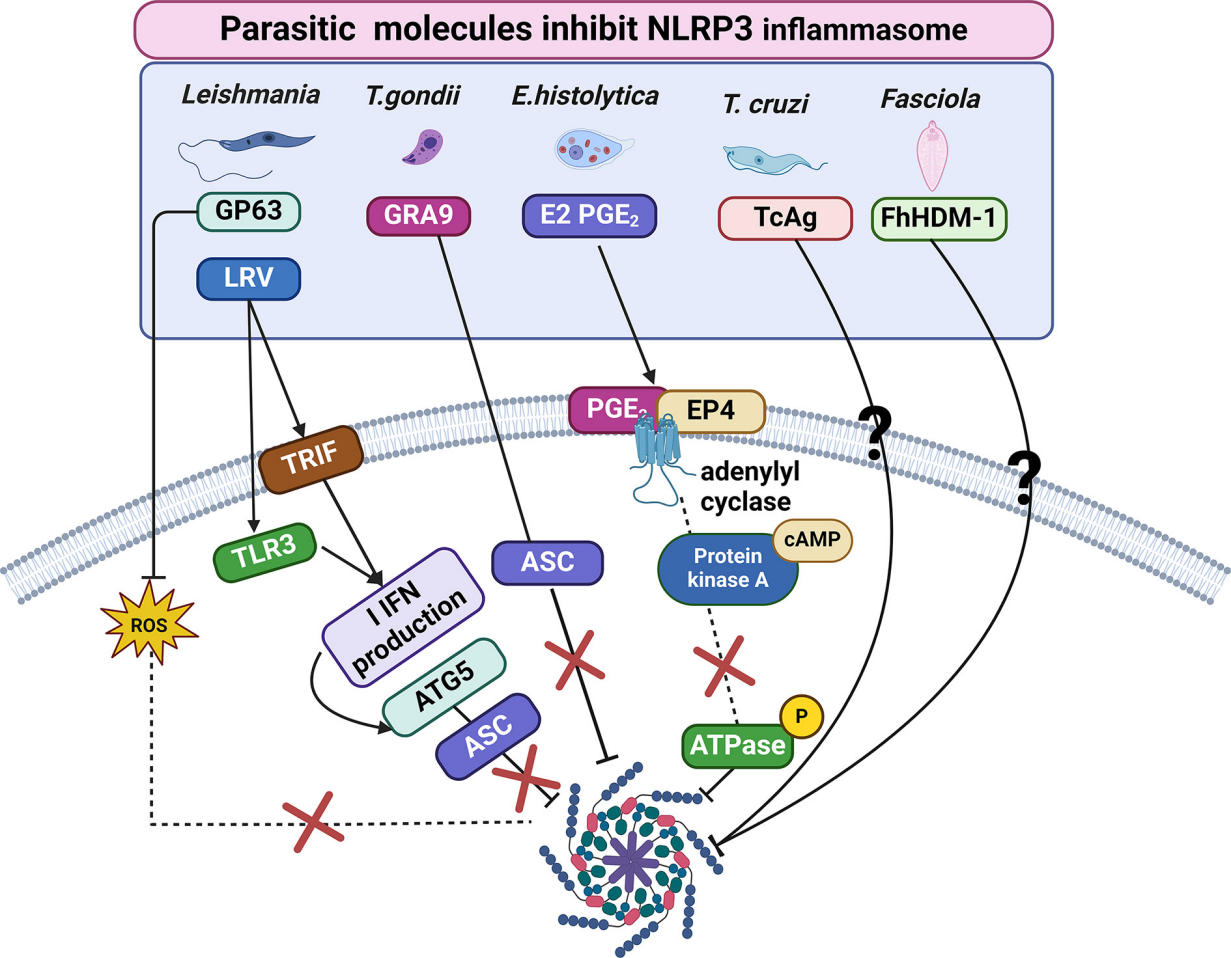
Schematic representation of the mechanisms of NLRP3 inhibition by parasitic molecules The virulence factor GP63, expressed by all Leishmania species inhibits reactive oxygen species (ROS), resulting in NLRP3 inflammasome suppression. While, Leishmania RNA virus (LRV) activates TLR3 and TRIF to produce type I IFN resulting in autophagy which induce the expression of Autophagy related 5 (ATG5), that able to block NLRP3 and apoptotic speck-containing (ASC) formation. Also, identically, the Dense granule protein 9 (GRA9) of *T. gondii* suppresses the NLRP3 inflammasome activation by blocking the binding of ASC-NLRP3 and causing disruption of the NLRP3 inflammasome formation. The *E. histolytica* lipid mediator prostaglandin E2 (PGE2) inhibits NLRP3 inflammasome via the PGE2 receptor; as a result of PGE2 signal transduction bonding with the EP4 receptor, adenylyl cyclase activated, subsequently increased intracellular level of cyclic adenylyl monophosphate (cAMP). The Protein Kinase A (PKA) mediates cAMP signaling to directly phosphorylate the Ser295 position of NLRP3 and prevent its ATPase function, resulting in NLRP3 oligomerization inhibition. *T. cruzi* antigen (TcAg) decreases NLRP3 inflammasome expression. Helminth defence molecule-1 of *F. hepatica* (FhHDM-1) (a cathelicidin-like peptide) suppresses NLRP3 inflammasome activation and reducing IL-1β secretion.

As discussed, different species of *Leishmania* can suppress the production of IL-1β both *in vitro* and *in vivo*. In this context, Shio et al. found that *L. mexicana* reduces IL-1β macrophage production through its virulence factor GP63 (the metalloprotease expressed by all *Leishmania* species). Also, the reduction of IL-1β production has been associated with the inhibition of reactive oxygen species (ROS) secretion, which has been linked to NLRP3 inflammasome activation. This ROS suppression is thought to result from damaged PKC-mediated protein phosphorylation. This finding indicates that the *Leishmania* surface GP63 molecule can significantly suppress NLRP3 inflammasome activation, resulting in a reduction of IL-1β production [89]. *Leishmania*, therefore, employs a unique protective mechanism to manipulate the host’s immune response. A subsequent study found that BALB/c mice infected with *L. donovani* produced IL-1β when given the antileishmanial drug Amp B. In contrast, administering the anti-IL-1β antibody to infected Amp B-treated mice increased the parasitic burden. This suggests that *Leishmania* is able to inhibit NLRP3 inflammasome activities, which in turn suppresses caspase-1 activation, and therefore IL-1β maturation, which is accompanying with reduction in NF-κB activity [90]. This study also used gene silencing of A20 (a negative regulator of NF-κB signaling) or UCP2 (mitochondrial uncoupling protein 2) in macrophages infected with *Leishmania* and concluded that *Leishmania* utilizes A20 and UCP2 to prevent inflammasome activation, resulting in their multiplication [90]. Furthermore, the *Leishmania* RNA virus (LRV) is a key virulence factor related to the progression of mucocutaneous leishmaniasis, a severe form of the disease [46]. A study that combined data from humans and animals revealed that LRV stimulates TLR3 and TRIF to trigger type I IFN production, resulting in autophagy. This leads to Autophagy-related 5 (ATG5) expressions which mediated the breakdown of NLRP3 and ASC, thus reducing NLRP3 inflammasome activation in macrophages [47]. Also, it is suggested that LRV inhibits caspase-11 activation and IL-1β production dependent on both TLR3 and ATG5. Therefore, this signaling pathway utilized by LRV is in the parasite’s favor by increasing its survival and pathogenicity [53]. It is clear that *Leishmania* develops several mechanisms to escape the host’s immune response by targeting NLRP3 inflammasome activation resulting in suppression of inflammatory response (Figure 3).

In summary, while the knockout mice studies indicate that NLRP3 activation during leishmaniasis is important for infection control, several studies have shown that the lack of NLRP3 also leads to a reduction in infection severity and mortality. It seems, therefore, that multiple factors, such as the parasite species and susceptibility of the host to infection, influence NLRP3 activation and lead to its dual action during *Leishmania* infection. Antileishmanial therapeutics will need greater research into the molecular pathophysiology of NLRP3 inflammasome activation in response to viral leishmaniasis.

#### Toxoplasma gondii

*Toxoplasma gondii* (*T. gondii*) is an intracellular parasitic organism able to infect all warm-blooded animals, including humans (where it infects about one-third of the global population) [91]. Most immune-competent individuals infected with *T. gondii* are asymptomatic or experience only mild and self-limiting illness [55]. However, extremely virulent strains of *T. gondii* can result in ocular disease in immune-competent adults [91]. Immunocompromised individuals may develop severe complications associated with *T. gondii* infection [55]. Infection during pregnancy with *T. gondii* is particularly serious as congenital toxoplasmosis may develop, resulting in abortion or neonatal mortality [54].

The immune response to *T. gondii* infection is complex due to the high level of heterogeneity in the genetic backgrounds of hosts, and the diverse virulence of parasite strains [92]. Immune responses during infection involve early production of proinflammatory cytokines, such as IL-12, to induce the production of IFN-γ by natural killer (NK) cells, CD4+ T cells, as well as CD8+ T cells [4]. IL-12 and IFN-γ are crucial in facilitating parasite death and controlling its growth [93]. The adaptive immunity against *T. gondii* infection involves maintaining a balance between the cell-mediated and humoral immune response actions of Th1 and Th2 cells. The Th1 response provides a strong protective role, characterized by activation of dendritic cells (DC) to produce IL-12 [94]. Th1 cells also produce IFN- γ as well as TNF-α cytokines, which stimulate the macrophages’ killing mechanisms against intraocular parasites [95].

Initiating the innate immune response is essential for controlling *T. gondii* infection. Limiting parasite proliferation appears to involve a defensive inflammasome-mediated response [96]. *In vitro*, infecting murine bone marrow-derived macrophages with *T. gondii* activates the NLRP3 inflammasome, causing an increase in IL-1β production. Furthermore, infecting knockout mice for NLRP3, caspase-1/11, IL-1R or the adaptor protein ASC, causes a reduction in IL-18 secretion and an increase in the parasitic burden that eventually leads to host death [72]. The activation of NLRP3 in a human fetal small intestinal epithelial infected with *T. gondii*, was mediated by P2X7R and resulted in IL-1 β production, and therefore inhibited *T. gondii* proliferation [97]. Infecting macrophages with *T. gondii* have been found to activate P2X7R and limit parasite proliferation. This P2X7R activation pathway involves the initiation of NADPH-oxidase-dependent ROS production, and activating an inflammasome, resulting in increased IL-1β secretion and ROS generation [98]. Furthermore, a study showed that NLRP3 was an inflammasome sensor activated during *T. gondii* infection in primary human peripheral blood cells, and its activation is mediated by the release of intracellular potassium [99]. It is suggested that *T. gondii* activates the NLRP3 inflammasome in primary human peripheral blood monocytes via the Syk-CARD9/MALT-1-NF-κB signaling pathway, resulting in IL-1β production [100].

Several studies explore the potential parasitic components that can impact NLRP3 activation during *T. gondii* infection. For instance, the soluble total Ag (STAg) derived from *T. gondii* strain RH, has been shown to stimulate NLRP3 activation and thereby increase IL-1β secretion *in vitro* [101]. A recent study revealed that the *T. gondii* secretory protein, rhoptry protein 7 (ROP7), can bond with the NACHT domain of NLRP3 in differentiated THP-1 cells, causing significant up-regulation in NF-κB expression and therefore inflammasome hyper activation via the IL-1β/NF-κB/NLRP3 pathway [75]. More recent study on THP-1 cell line treated with Profilin from *T. gondii* (TgP) reported that an increase in NLRP3 expression resulting in IL-1β production [102]. In contrast, Kim et al. found that Dense granule proteins 9 (GRA9), a secretory protein produced by *T. gondii*, is involved in disrupting the formation of the NLRP3 inflammasome. The protein blocks the binding of apoptotic speck-containing (ASC)-NLRP3, and suppresses the effect of NLRP3 [103]. In contrast, different *T. gondii* effector proteins, like GRA15, promote the NLRP3 inflammasome activation, resulting in IL-1β and IFN-γ production in THP-1 cells. This induces iNOS expression and NO secretion, causing the inhibition of IDO1 expression and therefore increased *T. gondii* growth in hepatocytes [104]. Additional effector proteins, such as GRA35, GRA42, and GRA43, also have a key role in *T. gondii* infection through pyroptosis stimulation and IL-1β production in Lewis’s rat BMDMs. However, whether such effector proteins have direct interactions with NLRP3 has not been proven [105] (Figure 3).

Regarding the involvement of inflammasome during the chronic stage of toxoplasmosis. Studies on the immune response to *T. gondii* at the chronic infection stage have found a vacuolar antigen of the parasite present in the host's macrophages. This suggests that proliferation of the parasite is controlled through a unique pathway involving NLRP3 induction of CD8 T cell IFN-γ responses [106].

The research discussed here indicates that *T. gondii* products are able to activate the NLRP3 inflammasome, which then produces IL-1β to control the infection. In contrast, the absence of it or any of its components, results in increased parasitic growth and mortality, implying that NLRP3 activation during toxoplasmosis serves a protective function. This review also enhances our understanding of the NLRP3 activation mechanism during *T. gondii* infection, data of value for the development of drugs to improve infection outcomes. However, further studies are required to understand the role of other parasitic products in the NLRP inflammasome’s activation.

#### Entamoeba histolytic

Amoebiasis is a parasitic disease that infects the large intestine of humans caused by an extracellular parasitic protozoan, *Entamoeba histolytica (E. histolytica)* [107]. According to the WHO, 500 million people worldwide are infected with Entamoeba; only 10% of these individuals are infected with *E. histolytica*, while the remaining are infected with non-pathogenic species like *Entamoeba dispar* and *Entamoeba coli*. Annually, amoebiasis can result in 40,000–100,000 deaths, which makes it the fourth protozoan infection causing death [108]. In general, the transmission route of *E. histolytica* to a host is by ingesting contaminated water or food due to faecal excretion of cysts or person-to-person contact [109]. *E. histolytica* is a virulent pathogen that is able to secrete molecules to break down and kill the host tissues and cells, in addition to engulfing red blood cells [107]. It infects the intestinal tract of humans, causing amoebiasis, which is clinically asymptomatic; however, an invasive host’s intestinal may result in the disease manifesting including abdominal pain, watery or bloody diarrhoea and weight loss [110]. In some cases, amoebas can breach the mucosal barrier of the intestine and travel to other organs, like the liver, lung, and, in some cases the brain, resulting in amoebic abscesses [60]. *E. histolytica* is predominantly found in the large intestine without initiating symptoms; however, in unknown conditions, the amoebae attack the mucosa and epithelium, causing intestinal amoebiasis, causing tissue lesions that progress to abscesses and a host acute inflammatory response [111].

Establishing an amoebic infection includes a critical balance between the parasite pathogenicity and immune response. Amoebas live in the outer mucus layer of the intestinal tract, where they can feed on gut bacteria. However, the reasons by which amoebas attack the host tissues are not completely known. After amoebas invade the tissues, the immune system triggers a response against the parasite [112]. Nevertheless, the key immune mechanisms against amoebas are still poorly understood [113]. Several studies with *E. histolytica* showed that trophozoites bind to TLR-2 and TLR-4 in human colonic cells through the carbohydrate recognition domain of the Galactose/N-acetylgalactosamine (Gal/GalNac) lectin and the lipopeptidophophoglycan (LPPG) located in the parasite surface. By acting as pathogen-associated molecular patterns (PAMPs), these amebic molecules trigger the classical TLR signaling pathway, prompting NFkB activation and increased expression of TLRs followed by inflammatory cytokines production [114]. That includes IL1β, IL-6, IL-8, IL-12, IFN-γ, and TNF-α, which further regulate the functions of the host immune response [115]. Furthermore, the secretion of *E. histolytica* macrophage migration inhibitory factor (MIF) (EhMIF) is vital for initiating the intestinal inflammation during amoebic invasion [116]. Macrophages are as well play a vital role in defence against amoebiasis via their production of a variety of inflammatory cytokines, such as IL-1β, IL-6, and IL-12 as well as NO, resulting in *E. histolytica* prolifration reduction [117–119]. As part of the innate response during amoebic infection Prostaglandin E2 (PGE2) of *E. histolytica*, induces the secretion of IL-8, a potent neutrophil chemoattractant [59]. An additional proinflammatory cytokine produced during amoebic infections is TNF-α, and its production is associated with *E. histolytica*-induced diarrhoea in children as well as tissue damage in the amoebic liver abscess in mouse models [56,120]. Therefore, it can be suggested that amoeba-induced inflammatory response results in tissue injury that can favor amoeba invasion.

Adaptive immunity also plays a significant role in the host defence against *E. histolytica*. A study in C3H mice infected with *E. histolytica* found that the diminution of CD4+ cells significantly reduced both parasite growth and inflammation, which also correlated with a decline in IL-4 and IL-13 production [121]. Thus, this study indicates that the importance of CD4+ T cells in mediating inflammation also contributes to the disease progress. Moreover, the type of cytokines produced from T cells might impact the disease outcome; for example, IFN-γ as a proinflammatory has a protection role during amebiasis via initiating the killing mechanisms of neutrophils and macrophages to control amoebicidal activity [45,84,96] (173). In contrast, IL-4 is an anti-inflammatory involved in the acute phase and during amoeba’s invasion [120,61,57]. IL-10 is an additional cytokine with a central protective role during intestinal amoebiasis by triggering resistance to intestinal amoebiasis in B6 mice [58]. Furthermore, CD8+ cytotoxic T cells can cause death to amoebas either directly or through the production of IL-17 [122]. However, Treg cells have been identified in a model of amoeba infection, and their role is characterized by participating in the control and resolution of the inflammatory response to *E. histolytica* infection [123]. Together, these studies suggest that cell-mediated immune responses have a significant contribution against *E. histolytica* infections. Therefore, the immune system-activated inflammation seems a double-edged sword: it can defend the host from *E. histolytica* invasive infection or stimulate severe tissue damage, facilitating *E. histolytica* distribution.

It was found that the NLRP3 inflammasome activation played a significant role during *E. histolytica* infection leading to IL-1β/IL-18 production and parasitic clearance from tissue [62]. Noticeable, NLRP3 inflammasome activation by *E. histolytica* does not trigger pyroptosis, which is a normal strategy of the host to remove intracellular parasites, as an alternative inflammasome can facilitate cell death leading to delay in the suppression of parasitic invasive, which can be unfavorable to innate host defences [63,106]. Following *E. histolytica* invasion into the lamina propria, macrophages immigrate to the site of infection and orchestrate robust inflammatory responses. This response includes priming and activating the NLRP3 inflammasome component genes, leading to the production of IL-1β/IL-18. It was found that Gal/GalNAc lectin can activate NF-κB and MAP kinase-signaling pathways in macrophages, resulting in up-regulation of the transcription of proinflammatory cytokines in addition to NLRP3 inflammasome components and pro-IL-1β [124]. A study in macrophages found that Gal/GalNAc is equivalent to LPS in up-regulating the pro-IL-1β and NLRP3 expression and achieves the priming requirements for NLRP3 activation, which all need NF-κB activation. Thus, this study suggested that Gal/GalNAc as soluble ligands may trigger TLRs in macrophages [63] (Figure 2).

Exceptionally, *E. histolytica*-prompted NLRP3 inflammasome activation includes direct interaction of intact live *E. histolytica* by Gal/GalNAc lectin-mediated binding [63]. As a result of NLRP3 activation, the recruitment and activation of caspase-1 will occur, causing the cleaving of the precursor IL-1β/IL-18 into their bioactive form. Several studies also found that the release and processing of IL-1β in response to *E. histolytica* is caspase-1 dependent, since inhibition of caspase-1 using specific inhibitors reduce the release of these proinflammatory cytokines [125]. Furthermore, the stimulating molecular mechanism of inflammasome and caspase-1 activation involves the formation of an intracellular bridge between cysteine proteinases containing an arginine-glycine-aspartate (RGD) binding with macrophages α5β1 integrin [62]. In supporting this, a study showed that when the Gal/GalNAc lectin interactions with macrophages, both α5β1 integrin and NLRP3 are enrolled into an intracellular junction, enabling EhCP-A5 RGD domain to directly cooperate with α5β1 integrin. Subsequently, this activation will trigger Src family kinase phosphorylation and opening of pannexin-1 (Panx1) channel to facilitate the rapid extracellular release of ATP. The free ATPs then return to macrophages via P2X7 receptors to initiate the second signal for NLRP3 inflammasome complex formation [126]. Therefore, *E. histolytica* is also able to induce NLRP3 activation in a two-signal event; the first signal involves the direct *E. histolytica* interaction that is facilitated by the Gal/GalNAc lectin forms an immune cell synapse to induce EhCP-A5 RGD linking with α5β1 integrin. The second signal occurs due to the extracellular ATP release acting in an autocrine manner via host P2X7 receptors to stimulate downstream signal transduction events. A study proposed that an EhCP-A5 RGD, expressed on the trophozoite surface and as secreted molecules, is essential for contact-dependent inflammasome activation [62]. Notably, *E. histolytica* can induce inflammasome activation via the efflux of potassium since the blocking of K+ channel activity causes IL-1β inhibition [127]. Also, Nlrp3−/− and Asc−/− mice showed reduced colonic production of IL-1β in response to live *E. histolytica* [62]. Li et al. also found that trophozoites abundantly secrete peroxiredoxins (Prx) during host cell invasion, and Prx C-terminal is considered as the main functional domain that can trigger NLRP3 in macrophage, and its activation pathway involves the binding of Prx with either TLR4 or P2X7 receptors [128]. These findings suggest that *E. histolytica* can trigger the activation of NLPP3 inflammasomes in a priming and activating fashion, resulting in an inflammatory response (Figure 2).

However, a study suggested that the lipid mediator prostaglandin E2 (PGE2) could modulate inflammatory response by inhibiting transcription of different proinflammatory genes such as IL-1β, TNF-α and IL-8, therefore suppressing NLRP3 inflammasome [62]. Mechanically, following PGE2 signal transduction coupling with the EP4 receptor, adenylyl cyclase will be activated, resulting in up-regulated intracellular concentration of cyclic adenylyl monophosphate (cAMP) [129]. Then, the Protein Kinase A (PKA), the key mediator of cAMP signaling kinase, directly phosphorylates the Ser295 position of NLRP3 and turns off its ATPase activities [62]. Therefore, the self-oligomerization of NLRP3 and the inflammasome complex assembly are inhibited [130] (Figure 3). Thus, the Production of PGE2 plays a crucial role in disease pathogenesis and immune evasion during *E. histolytica* via inhibition of inflammasome activation. However, the mechanism of how this inhibition occurs and whether this is beneficial for *E. histolytica* are not yet clear.

Collectively, it has become well understood that as an extracellular parasite, *E. histolytica* can activate the NLRP3 inflammasome complex via direct interaction with the live parasite and macrophages. However, intercellular interaction also can include the activation of NLRP3 via Gal/GalNAc mediated adherence with macrophages, facilitating an intercellular bridge between EhCP-A5 and α5β1 integrin, resulting in the rapid extracellular release of ATP that will eventually activate the NLRP3 inflammasome. Although PGE2 suppresses the activation of NLPR3, the actual immunological impact of this inhibition on either the host or parasite is still vague. It is clear that NLRP3 activation is one of the vital innate events during *E. histolytica*, resulting in an inflammatory response that will control the infection’s progression. However, compared with another parasitic infection discussed here, it is noticeable that most studies regarding the NLRP3 inflammasome*-E.histolytica* interaction were *in vitro* studies. Thus, supplementary *in vivo* studies are required to evaluate the interaction between the parasite and the host’s immune response within the complex network of biological influence and cross-regulatory pathways during amoebiasis. Like the influence of *E. histolytica* bioenvironmental, in which colonic cells are typically exposed to pathogenic and commensal organisms within the colon. Also, it is crucial to understand the overall impact of inflammasome activation or inhibition on either the host response or infection progression. Therefore, it can be proposed that the NLRP3 inflammasome action needs further investigation by applying additional *in vivo* experiments, including NLRP3 knockout animal models. Knowing the immunological functions of NLRP3 during amoebiasis will benefit in preventing the disease pathogenesis of *E. histolytica* infection.

#### Trypanosome cruzi

*Trypanosome cruzi* is the parasite that causes Chagas disease, a potentially fatal infection that can affect the heart and gastrointestinal tract [131]. The WHO estimates that Chagas disease is the most important parasitic disease in the Americas, accounting for five times as many infections as malaria. In 2015, WHO estimated that 7 million people were infected, the majority living in Latin America, with 25 million at high risk of contracting the chronic form of the disease [132]. The spread of Chagas disease beyond the geographical areas it was once confined to, has transformed it into a global healthcare issue [133]. *T. cruzi* is normally found in the guts of hematophagous triatomine bugs, and transmission occurs when infected bug faeces contaminate the bite site or mucous membranes of the host. *T. cruzi* can also be transmitted by transfusion, tissue transplants, and congenitally [134,135]. *T. cruzi* strains are classified into seven different type units (DTUs), TcI toTcVI and TcBat, whose virulence, and pathogenicity in the vertebrate host, differ greatly [136]. Most patients are asymptomatic or have mild or nonspecific symptoms such as fever [135]. However, 1% of patients develop severe acute disease (Chagas disease), with potentially fatal symptoms that include acute myocarditis, pericardial effusion, and meningoencephalitis [134,137].

To establish chronic infection, *T. cruzi* triggers a complex response in the host immune system [138]. Experimental models have shown that *T. cruzi* surface glycoproteins (mucins) and/or glycophospholipids (GIPLs), can activate the innate immune cells to produce IFN-γ, TNF-α, IL-1β, and IL-6. These cytokines stimulate the production of NO and superoxide by macrophages which cause parasite death [135]. When mice are infected with *T. cruzi*, IFN-γ and IL-12 trigger protective adaptive immunity, including the parasite-specific Th1 response [139]. Generally, it is found that the resistance against acute experimental *T. cruzi* infection involves the activation of several innate immune receptors, such as Toll-like and Nod-like receptors, and the NLRP3 inflammasome [140].

As previously established, NLRP3 is an essential immunological component during *T. cruzi* infection. A study in mice evaluated the influence of *T. cruzi* virulence (low, medium, high) on the expression of several innate immune mediators, including NLRP3, and concluded that highly virulent *T. cruzi* strains up-regulate the expression of NLRP3, caspase-1, IL-1β and iNOS mRNA in heart muscle more than strains with low or medium virulence. These effects may be responsible for the myocarditis and increased mortality associated with some *T. cruzi* infections [141]. A study by Goncalves et al. demonstrated that *T. cruzi* infection triggers IL-1β production in an NLRP3- and caspase-1-dependent manner in peritoneal macrophages (PMs), and that cathepsin-B was required for the activation of NLRP3. Importantly, NLRP3-/- and caspase1-/- mice were found to host more *T. cruzi* parasites than MyD88-/-and iNOS-/-mice (which are susceptible models for *T. cruzi* infection), showing that the NLRP3 inflammasome contributes to acute infection control. In addition, when these NLRP3 and caspase-1 knockout mice were infected with *T. cruzi*, they decreased NO production and limited macrophage-mediated parasite killing [142]. These data demonstrate how the activation of NLRP3, and subsequent NO production, functions as a unique effector-killing mechanism to control *T. cruzi* infections. As described earlier, in 1% of patients *T. cruzi* infection develops into Chagas disease, which may result in life-threatening meningoencephalitis. Of particular relevance to this aspect of *T. cruzi* infection, one study found that NLRP3 is activated within the microglia. This activation results in IL-1β and NO secretion which contributes to the pathogenesis of *T. cruzi* infection within the CNS [143].

It appears that NLRP3 has a critical function in regulating infection, hence various research has been undertaken to fully understand its activity. One mechanism proposed for the activation of the ASC/NLRP3 pathway by *T. cruzi*, includes K^+^ efflux, lysosomal acidification, ROS production and lysosomal impairment. One study also observed that ASC and caspase-1 knockout mice infected with *T. cruzi* had higher mortality and heart inflammation, suggesting that inflammasomes play a critical role in host resistance to the parasite [144]. Another study utilized wild-type (WT), ASC -/-, and NLRP3 -/- macrophages, as well as human macrophages, and suggested that *T. cruzi* infection provokes delayed activation of inflammatory cytokine gene expression and IL-1β production in NLRP3 -/- macrophages. However, these macrophages showed significant reductions in intracellular parasite proliferation compared to WT controls. This study also found that caspase-1/ASC inflammasomes play a key role in the activation of IL-1β /ROS and NF-kB signaling of cytokine gene expression in human and mouse macrophages, which contributes to the control of *T. cruzi* infection [145]. Research in human THP monocyte-derived macrophages found *T. cruzi* to strongly suppress TXNIP expression, an antioxidant inhibitor that facilitates caspase-1 activation upon recruitment to NLRP3 inflammasome [146]. Furthermore, rapamycin-pretreated macrophages infected with *T. cruzi* have been found to show much greater NLRP3 and mitochondrial ROS (mtROS) expression compared with control cells. However, when mtROS production was inhibited in rapamycin-pretreated infected macrophages from NLRP3 KO mice, the parasitic replication significantly increased. Suggesting that mTOR suppression during *T. cruzi* infection triggers NLRP3 activation and mtROS production, causing macrophage inflammatory response that regulates *T. cruzi* proliferation [147]. These data suggest that NLRP3 inflammasome activation can be induced by *T. cruzi* resulting in inhibition of mTOR production and consequent limiting of parasite replication. The activation of the inflammasome is a specific strategy that necessarily influences inflammatory outcomes.

It is clear that macrophages are one of the major cell types mediating the recognition and modulation of immune responses during *T. cruzi* infection. For example, *T. cruzi* can facilitate the macrophage galactose-C type lectin (MGL 1) receptor to initiate the innate immune response. Stimulating MGL1 knockout macrophages *in vitro* with *T. cruzi* antigen (TcAg) has been shown to reduce procaspase-1, caspase-1, and NLRP3 inflammasome expression [148]. (Figure 3). This finding reveals a possible mechanism for the NLRP3 activation pathway in macrophages during the immune response to *T. cruzi*. IL-1β production by macrophages is crucial for T-cell activation during *T. cruzi* infection. Paroli et al. investigated the role of NLRP3 and caspase-1/11 in the differentiation and activation of T cells during acute infection with a *T. cruzi-Tulahuen* strain [149]. They found that during infection, NLRP3−/− and C57BL/6 WT mice showed similar parasitemia and survival rates, although the parasite burden was greater in the livers of NLRP3−/− mice than WT mice. Suggesting that NLRP3 is not needed for regulating parasitemia, but is still crucial for improved parasite clearance from the liver. Importantly, they found that the differentiation of T helper and cytotoxic T lymphocyte phenotypes depended on whether the mice were deficient in NLRP3 or caspase-1/11. Notably, caspase-1/11−/− mice showed a significant decrease in the number of IFN-γ- and IL-17-producing CD4+ and CD8+ T cells, which are linked to higher parasite loads and lower survival [149]. These results imply that NLRP3 pathway activation is vital for assembling an appropriate T cell response during *T. cruzi* infection.This finding reveals a possible mechanism for the NLRP3 activation pathway in macrophages during the immune response to *T. cruzi*. Autophagy is one of the effector mechanisms that limit *T. cruzi* infection. For example, a study showed that NLRP3 is needed to stimulate an autophagic flux during *T. cruzi* infection by mediating the autolysosome formation in peritoneal macrophages (PMs) from C57BL/6 WT mice, thereby limiting *T. cruzi* replication [148].

It is obvious that NLRP3 inflammasome activation is influenced by the strain of *T. cruzi* during the infection course. In addition, the NLRP3 knockout mice studies show that lacking this inflammasome significantly affects the macrophage-mediated parasite-killing mechanism, resulting in heart inflammation and higher mortality. NLRP3 deficiency also has an impact on the development of T cell responses during *T. cruzi* infection by reducing CD4+ and CD8+ T cell numbers, leading to higher parasite loads and lower survival. Inflammasome activation may contribute to inflammatory responses during *T. cruzi* infection, through its inhibitory effect on mTOR production, which reduces parasite growth. However, no studies have examined the potential role of parasitic surface proteins, such as Mucin and Trans-Sialidase, in either NLRP3 inflammasome activation or inhibition. NLRP3 activation may therefore have other functions during *T. cruzi* infection which remain to be discovered. Understanding these key immunological cellular pathways will help to develop drugs for controlling *T. cruzi* infection and limiting the immunopathology of Chagas disease.

#### Helminths

Helminths are complex, multicellular, parasitic worms occupying a wide range of geographical, ecological, and anatomical niches, and with highly complex life cycles. Helminths are categorised into three classes: nematodes (roundworms), platyhelminths (flatworms, including trematodes and cestodes), and annelids (segmented worms, including leeches) [150]. It is estimated that approximately 2 billion individuals are infected with the parasite, making it the most common human infection in developing countries [151]. Moreover, helminths have several invasion routes, including the skin (schistosomes and hookworms) and mosquito bite (filarial worms), but the most common is via the gastrointestinal tract [152]. The disease in humans is normally caused by adult worms, egg deposition in tissues, or migration of larvae or microfilariae. Helminth infections are normally asymptomatic or mild, but immunologically naïve and immunosuppressed individuals can experience severe clinical outcomes [150].

Helminths can form long-term chronic infections during which the host immune response is severely suppressed [153]. The remarkable distribution of helminth infections arises from their ability to manipulate the host immune system by controlling its susceptibility, resistance, and pathogenesis [154]. Although protective immunity to helminths in humans is not well understood, animal models of infection have indicated that human immunity is mediated by the Th2 response [154,155]. The latter seems to be targeted by the helminth immunoregulation mechanism as a means to establish a successful opportunistic parasite–host relation [152]. Asymptomatic infection shows increased production of anti-inflammatory cytokines such as IL-10 and high levels of circulating T cells expressing the inhibitory marker CTLA-4 (cytotoxic T lymphocyte antigen 4) [156,157]. There is also inhibited production of Th1 inflammatory cytokines, such as IFN-γ [158]. However, in severe and deteriorating cases, lymphatic pathology develops with fewer regulatory T cells and increased Th1 and Th17 effector responses – which might explain the severe lymphatic inflammation outcome [159]. The relationship between these parasites and the host immune response is highly complex, and a full analysis is beyond the scope of this review.

Activation of the NLRP3 inflammasome plays a key role in helminth infections by provoking Th2 and Th17/inflammatory responses [160,161]. Potential stimuli for NLRP3 activation during infections are helminth products that are either soluble or exosomal, and endogenous signals from inflammation and injured tissue [161]. One study found that soluble schistosomal egg antigens (SEA) can activate the NLRP3 inflammasome, resulting in IL-1β production in dendritic cells. SEA protein appears to function as a second signal for inducing proteolytic pro-IL-1β cleavage (Figure 2). Moreover when mice deficient in the central inflammasome adapter ASC, but had NLRP3 molecules infected with *Schistosoma mansoni*, they showed a reduction in IL-1β expression and liver immunopathology [162]. In contrast, infecting WT mice with *Schistosoma japonicum* (*S. japonicum*) resulted in high expression of IL-1β, and NLRP3 activation. In examining this activation mechanism, Meng et al. observed that hepatic mouse stellate cells (HSCs) cultured with soluble egg antigen, induced NLRP3 inflammasome formation, which was linked to both redox regulation and lysosomal dysfunction [163]. This suggests that NLRP3 inflammasome activation plays a role in initiating the inflammatory action that leads to liver fibrosis associated with *S. japonicum* infection. Several earlier studies have shown that the inflammatory action of NLRP3 inflammasomes during schistosomiasis in the liver could be limited by taurine (a sulfur-containing β-amino acid) [164]. In mice infected with *S. japonicum*, taurine was found to suppress activation of the hepatic thioredoxin-interacting protein (TXNIP)/NLRP3 inflammasome, thereby preventing IL-1β production and pyroptosis. The study also found that NLRP3-deficient mice infected with *S. japonicum*, developed hepatosplenomegaly, liver dysfunction, hepatic granulomas, and fibrosis, and showed reduced NLRP3-dependent liver pyroptosis. The authors suggest that taurine’s ability to control the activation of the TXNIP/NLRP3 inflammasome pathway might make it an effective preventative of liver pathology during *S. japonicum* infection [165].

Many studies examine the immunological action of NLRP3 during trematode infection. It is found that the FhCL3 helminth-derived molecules of *Fasciola hepatica* can induce non-canonical inflammasome activation in dendritic cells (DCs), resulting in IL-1β and IL-18 production, and this has been associated with the cysteine protease activity of FhCL3 – an independent caspase pathway. The activation of the NLRP3 inflammasome by FhCL3, prompts the adaptive immune response and is characterized by the secretion of IFN-γ and IL-13 [166]. These data indicate that the helminth-derived molecule FhCL3, can activate the NLRP3 inflammasome in a caspase-independent manner. However, Alvarado et al. demonstrated that NLRP3 inflammasome activation can be inhibited by Helminth defence molecule-1 of *F. hepatica* (FhHDM-1) (a cathelicidin-like peptide), resulting in a reduction in IL-1β secretion by macrophages (Figure 3). The inhibitory outcome was associated with lysosomal cathepsin B protease causing IL-1β production and effective Th1 response suppression, eventually parasite survival [167]. Moreover, infected NLRP3−/− mice with *Trichinella spiralis*, have been shown to host more larvae than WT mice. In supporting the finding, administration of WT mice with MLES (muscle larvae excretory-secretory products) showed higher levels of IL-4, IL-10, TGF-β, and Tregs population, than NLRP3−/− mice receiving the same treatment. This was carried out *in vitro* by treating WT-DCs with MLES, and resulted in up-regulation of CD40 expression and increased production of IL-4, IL-10, TGF-β, and Tregs populations. Conversely, treating NLRP3 knockout cells with MLES, caused down-regulation of CD40 expression with increased production of IL-1β, IL-18, IL-10, and TGF-β, but not IL-12p70 [168]. This study explained the vital role NLRP3 plays in developing the Th2 and Treg responses of the host defence against *Trichinella spiralis*.

Although NLRP3 inflammasome activation seems to be important to host defences against helminth infections by regulating Th2 and Th17 responses, it can also cause uncontrolled inflammatory action that leads to liver immunopathology. This is confirmed by the NLRP3 knockout studies, where mice lacking the NLRP3 molecule had better disease outcomes. For instance, NLRP3-deficient mice infected with *Schistosoma japonicum* had reduced NLRP3-dependent liver pyroptosis. The absence of NLRP3 could also be favorable to parasite growth, as was indicated in the *Trichinella spiralis* infection studies. That the NLRP3 inflammasome appears to play a dual role in host defences against helminth infection might be due to the complexity of the parasite’s life cycle. Currently, there are too few studies to fully determine the role of NLRP3 or its activation mechanism, in the host response to helminth infections. Exploring these areas would therefore be important for a fuller understanding of this inflammasome’s contribution to anti-parasitic immune responses.

## Conclusion and future perspective

The NLRP3 inflammasome has many effects on the host response during parasitic infection. In some cases, it successfully fulfils its immunological role and protects the host. In others, however, its immunological response may be counterproductive, damaging the host or advantaging the parasite’s growth. Since NLRP3 inflammasome activation was found to exert significant control over *Leishmania, T.gondii* and *T. cruzi* infections. In addition, *E. histolytica* as extracellular can stimulate the NLRP3 inflammasome activation via outside-in signaling independent of pyroptosis leading to an inflammatory response against the parasite. Conversely, NLRP3 deficiency is also beneficial to the host, as it limits the infection severity in malaria and leishmaniasis, while its absence affects the *T. cruzi*-killing mechanism of macrophages and the differentiation of T-cell responses, resulting in greater parasite burdens. It is demonstrated that NLRP3 activation during helminth infection helps to control the parasite by triggering Th2 and Th17 responses. However, for some types of helminth species, a lack of NLRP3 can also reduce the parasite burden carried by the host.

This review suggests that different parasitic products might have different effects on the NLRP3 activation, and in some cases these effects could conflict, thereby accounting for the inflammasome’s contrary influences. It has been found that plasmodium products like Hz and DNA are capable of stimulating NLRP3 activation. Yet, further studies are needed to determine the potential role of other plasmodium molecules like GPIs and immunostimulatory DNA, in the NLRP3 function. Since the ability of GPIs to activate NF-kB signaling through TLRs resulting in the production of proinflammatory cytokines particularly IL-1β, proposing that GPIs might possibly interact with NLPR3 inflammasomes [162]. However, further investigation is needed to prove this point. Several Leishmania molecules and their mechanical actions have also been reported in this review, including LPG, which can stimulate NLRP3 activation, whereas GP63 and LRV both suppress it [35,52,76]. In addition, *T.gondii* products are found to be involved in the activation of NLRP3, such as STAg and ROP7 [50,163]. Conversely, GRA9 proteins show an anti-inflammatory response by suppressing NLRP3 formation [55]. In the case of *E. histolytica* the adherence molecules like Gal/GalNAc and EhCP-A5 RGD together are able to mediate NLRP3 inflammasome activation, while the production of PGE2 by the parasite indirectly inhibits it [114,62]. As earlier remarked, compared to other parasitic infections, very few studies have been carried out on the interaction of NLRP3 inflammasomes with *T. cruzi* and helminths molecules (Table 1).

The evidence here suggests that the NLRP3 inflammasome's interaction with parasites and their molecules *in vivo* remains only preliminary and requires further confirmation. It has been proposed that whether the NLRP3 inflammasome is activated or inhibited during infection depends on the parasite and the host’s genetic background. The host immune response, and the parasites’ regulation of that response, are vital areas that must be studied to attain the knowledge necessary to develop effective vaccines and treatment approaches to control these infectious diseases. In addition, in this increasingly advanced field, this review may have further new ideas about parasitic molecules' influence on inflammasome actions that provide clear clinical opportunities to develop new therapeutic interventions to treat these diseases.

## Abbreviations

AIM2: absent-in-melanoma 2
ASC: apoptosis-associated speck-like protein containing a CARD
ATG5: autophagy related 5
CARD9: caspase Recruitment Domain Containing Protein 9
CLRs: C-type lectin receptors
COX: cyclooxygenase
CP5: cysteine protease 5
DAMP: danger-associated molecular pattern
EhCP-A5 RGD: *E. histolytica* cysteine 5 proteinases contain an arginine-glycine-aspartate
EhMIF: *E. histolytica* migration inhibitory factor
EP4: prostaglandin EP4 receptor
FhCL3: *F. hepatica* cathepsin L3
FhHDM-1: *F. hepatica* Helminth defense molecule-1
Gal/GalNAc: galactose/ N-acetylgalactosamine
gDNA: genomic DNA
GIPL: glycophospholipid
GP63: glycoprotein-63
GPI: glycosylphosphatidylinositol anchor
GRA9: dense granule protein 9
Hz: Hemozoin
IRF3: interferon regulatory factor 3
LCL: localized cutaneous leishmaniasis
LmSd: leishmania major Seidman strain
LPG: leishmania glycoconjugate lipophosphoglycan
LPPG: lipopeptidophosphoglycan
LRR: leucine-rich repeat
LTB4: leukotriene B4
Lyn: LYN proto-oncogene, Src family tyrosine kinase
MIF: migration inhibitory factor
MLES: muscle larvae excretory-secretory
NACHT: nucleoside-triphosphatase
NLR: nucleotide-binding domain and leucine-rich repeat
NLRC4: NLR family CARD domain containing 4
NLRP3: NLR family pyrin domain containing 3
NLRP4: NLR family pyrin domain containing 4
NLR: NOD-like receptor
NOD: nucleotide-binding oligomerization domain
P2X7R: purinergic receptor P2X 7
PAMPs: pathogen-associated molecular patterns
Panx1: pannexin-1
PGE2: prostaglandin E2
PRR: pattern recognition receptor
Prx: peroxiredoxins
PYD: pyrin domain
RLRs: rig-I-like receptor
ROP7: rhoptry protein 7
SEA: schistosomal egg antigens
SOCS1: suppressor of cytokine signaling 1
Src: proto-oncogene tyrosine-protein kinase
STAg: soluble total Ag
TcAg: *T. cruzi* antigen
TgP: profilin from *T. gondii*
TLR: toll-like receptor

## References

[1] Pétrilli V., Dostert C., Muruve D.A. and Tschopp J. (2007) The inflammasome: a danger sensing complex triggering innate immunity. Curr. Opin. Immunol. 19, 615–622 10.1016/j.coi.2007.09.002 17977705

[2] Kawai T. and Akira S. (2009) The roles of TLRs, RLRs and NLRs in pathogen recognition. Int. Immunol. 21, 317–337 10.1093/intimm/dxp017 19246554 PMC2721684

[3] Miao E.A., Rajan J.V. and Aderem A. (2011) Caspase‐1‐induced pyroptotic cell death. Immunol. Rev. 243, 206–214 10.1111/j.1600-065X.2011.01044.x 21884178 PMC3609431

[4] Franchi L., Eigenbrod T., Muñoz-Planillo R. and Nuñez G. (2009) The inflammasome: a caspase-1-activation platform that regulates immune responses and disease pathogenesis. Nat. Immunol. 10, 241–247 10.1038/ni.1703 19221555 PMC2820724

[5] Sharma D. and Kanneganti T.-D. (2016) The cell biology of inflammasomes: Mechanisms of inflammasome activation and regulation. J. Cell Biol. 213, 617–629 10.1083/jcb.201602089 27325789 PMC4915194

[6] Lamkanfi M. and Dixit V.M. (2014) Mechanisms and functions of inflammasomes. Cell 157, 1013–1022 10.1016/j.cell.2014.04.007 24855941

[7] Thomas P.G., Dash P., Aldridge J.R. Jr, Ellebedy A.H., Reynolds C., Funk A.J. et al. (2009) The intracellular sensor NLRP3 mediates key innate and healing responses to influenza A virus via the regulation of caspase-1. Immunity 30, 566–575 10.1016/j.immuni.2009.02.006 19362023 PMC2765464

[8] Allen I.C., Scull M.A., Moore C.B., Holl E.K., McElvania-TeKippe E., Taxman D.J. et al. (2009) The NLRP3 inflammasome mediates in vivo innate immunity to influenza A virus through recognition of viral RNA. Immunity 30, 556–565 10.1016/j.immuni.2009.02.005 19362020 PMC2803103

[9] Gross O., Poeck H., Bscheider M., Dostert C., Hannesschläger N., Endres S. et al. (2009) Syk kinase signalling couples to the Nlrp3 inflammasome for anti-fungal host defence. Nature 459, 433–436 10.1038/nature07965 19339971

[10] Kanneganti T.-D., Body-Malapel M., Amer A., Park J.-H., Whitfield J., Franchi L. et al. (2006) Critical role for Cryopyrin/Nalp3 in activation of caspase-1 in response to viral infection and double-stranded RNA. J. Biol. Chem. 281, 36560–36568 10.1074/jbc.M607594200 17008311

[11] Zhong Y., Kinio A. and Saleh M. (2013) Functions of NOD-like receptors in human diseases. Front. Immunol. 4, 333 10.3389/fimmu.2013.00333 24137163 PMC3797414

[12] Menu P. and Vince J. (2011) The NLRP3 inflammasome in health and disease: the good, the bad and the ugly. Clin. Exp. Immunol. 166, 1–15 10.1111/j.1365-2249.2011.04440.x 21762124 PMC3193914

[13] Guo H., Callaway J.B. and Ting J.P. (2015) Inflammasomes: mechanism of action, role in disease, and therapeutics. Nat. Med. 21, 677–687 10.1038/nm.3893 26121197 PMC4519035

[14] Rada B., Park J.J., Sil P., Geiszt M. and Leto T.L. (2014) NLRP3 inflammasome activation and interleukin-1β release in macrophages require calcium but are independent of calcium-activated NADPH oxidases. Inflamm. Res. 63, 821–830 10.1007/s00011-014-0756-y 25048991 PMC4162906

[15] Zahid A., Li B., Kombe A.J.K., Jin T. and Tao J. (2019) Pharmacological inhibitors of the NLRP3 inflammasome. Front. Immunol. 10, 2538 10.3389/fimmu.2019.02538 31749805 PMC6842943

[16] Franchi L., Warner N., Viani K. and Nuñez G. (2009) Function of Nod‐like receptors in microbial recognition and host defense. Immunol. Rev. 227, 106–128 10.1111/j.1600-065X.2008.00734.x 19120480 PMC2679989

[17] Vajjhala P.R., Mirams R.E. and Hill J.M. (2012) Multiple binding sites on the pyrin domain of ASC protein allow self-association and interaction with NLRP3 protein. J. Biol. Chem. 287, 41732–41743 10.1074/jbc.M112.381228 23066025 PMC3516722

[18] de Zoete M.R., Palm N.W., Zhu S. and Flavell R.A. (2014) Inflammasomes. Cold Spring Harbor Perspect. Biol. 6, a016287 25324215 10.1101/cshperspect.a016287 PMC4292152

[19] Mamantopoulos M., Ronchi F., Van Hauwermeiren F., Vieira-Silva S., Yilmaz B., Martens L. et al. (2017) Nlrp6-and ASC-dependent inflammasomes do not shape the commensal gut microbiota composition. Immunity 47, 339.e4–348.e4 10.1016/j.immuni.2017.07.011 28801232

[20] WHO I. (2021) World malaria report 2017, World Health Organization, Geneva

[21] Bauernfeind F.G., Horvath G., Stutz A., Alnemri E.S., MacDonald K., Speert D. et al. (2009) Cutting edge: NF-κB activating pattern recognition and cytokine receptors license NLRP3 inflammasome activation by regulating NLRP3 expression. J. Immunol. 183, 787–791 10.4049/jimmunol.0901363 19570822 PMC2824855

[22] Liu T., Zhang L., Joo D. and Sun S.-C. (2017) NF-κB signaling in inflammation. Signal Transd. Targeted Ther. 2, 1–9 10.1038/sigtrans.2017.23 PMC5661633 29158945

[23] Francis S.E., Sullivan D.J. Jr and Goldberg D.E. (1997) Hemoglobin metabolism in the malaria parasite Plasmodium falciparum. Annu. Rev. Microbiol. 51, 97–123 10.1146/annurev.micro.51.1.97 9343345

[24] Ozaki E., Campbell M. and Doyle S.L. (2015) Targeting the NLRP3 inflammasome in chronic inflammatory diseases: current perspectives. J. Inflammation Res. 8, 15 10.2147/JIR.S51250 PMC4303395 25653548

[25] Franchi L., Eigenbrod T., Muñoz-Planillo R., Ozkurede U., Kim Y.-G., Chakrabarti A. et al. (2014) Cytosolic double-stranded RNA activates the NLRP3 inflammasome via MAVS-induced membrane permeabilization and K+ efflux. J. Immunol. 193, 4214–4222 10.4049/jimmunol.1400582 25225670 PMC4185247

[26] Latz E., Xiao T.S. and Stutz A. (2013) Activation and regulation of the inflammasomes. Nat. Rev. Immunol. 13, 397–411 10.1038/nri3452 23702978 PMC3807999

[27] Jo E.-K., Kim J.K., Shin D.-M. and Sasakawa C. (2016) Molecular mechanisms regulating NLRP3 inflammasome activation. Cell. Mol. Immunol. 13, 148–159 10.1038/cmi.2015.95 26549800 PMC4786634

[28] Kelley N., Jeltema D., Duan Y. and He Y. (2019) The NLRP3 inflammasome: an overview of mechanisms of activation and regulation. Int. J. Mol. Sci. 20, 10.3390/ijms20133328 31284572 PMC6651423

[29] Martinon F., Burns K. and Tschopp J. (2002) The inflammasome: a molecular platform triggering activation of inflammatory caspases and processing of proIL-β. Mol. Cell. 10, 417–426 10.1016/S1097-2765(02)00599-3 12191486

[30] Kayagaki N., Warming S., Lamkanfi M., Walle L.V., Louie S., Dong J. et al. (2011) Non-canonical inflammasome activation targets caspase-11. Nature 479, 117–121 10.1038/nature10558 22002608

[31] Kayagaki N., Wong M.T., Stowe I.B., Ramani S.R., Gonzalez L.C., Akashi-Takamura S. et al. (2013) Noncanonical inflammasome activation by intracellular LPS independent of TLR4. Science 341, 1246–1249 10.1126/science.1240248 23887873

[32] Kim J.-J. and Jo E.-K. (2013) NLRP3 inflammasome and host protection against bacterial infection. J. Korean Med. Sci. 28, 1415–1423 10.3346/jkms.2013.28.10.1415 24133343 PMC3792593

[33] Clay G.M., Sutterwala F.S. and Wilson M.E. (2014) NLR proteins and parasitic disease. Immunol. Res. 59, 142–152 10.1007/s12026-014-8544-x 24989828 PMC5106291

[34] Florens L., Washburn M.P., Raine J.D., Anthony R.M., Grainger M., Haynes J.D. et al. (2002) A proteomic view of the Plasmodium falciparum life cycle. Nature 419, 520–526 10.1038/nature01107 12368866

[35] Garrido-Cardenas J.A., González-Cerón L., Manzano-Agugliaro F. and Mesa-Valle C. (2019) Plasmodium genomics: an approach for learning about and ending human malaria. Parasitol. Res. 118, 1–27 10.1007/s00436-018-6127-9 30402656

[36] Rossi M. and Fasel N. (2018) How to master the host immune system? Leishmania parasites have the solutions! Int. Immunol. 30, 103–111 10.1093/intimm/dxx075 29294040 PMC5892169

[37] Su X.-Z., Lane K.D., Xia L., Sá J.M. and Wellems T.E. (2019) Plasmodium genomics and genetics: new insights into malaria pathogenesis, drug resistance, epidemiology, and evolution. Clin. Microbiol. Rev. 32, e00019–19 10.1128/CMR.00019-19 31366610 PMC6750138

[38] Martiney J.A., Sherry B., Metz C.N., Espinoza M., Ferrer A.S., Calandra T. et al. (2000) Macrophage migration inhibitory factor release by macrophages after ingestion of Plasmodium chabaudi-infected erythrocytes: possible role in the pathogenesis of malarial anemia. Infect. Immun. 68, 2259–2267 10.1128/IAI.68.4.2259-2267.2000 10722628 PMC97412

[39] Trampuz A., Jereb M., Muzlovic I. and Prabhu R.M. (2003) Clinical review: Severe malaria. Crit. Care 7, 315 10.1186/cc2183 12930555 PMC270697

[40] Weidanz W.P. (1982) Malaria and alterations in immune reactivity. Br. Med. Bull. 38, 167–172 10.1093/oxfordjournals.bmb.a071754 7052198

[41] Deroost K., Pham T.-T., Opdenakker G. and Van den Steen P.E. (2015) The immunological balance between host and parasite in malaria. FEMS Microbiol. Rev. 40, 208–257 10.1093/femsre/fuv046 26657789

[42] Ataide M.A., Andrade W.A., Zamboni D.S., Wang D., Souza Mdo C., Franklin B.S. et al. (2014) Malaria-induced NLRP12/NLRP3-dependent caspase-1 activation mediates inflammation and hypersensitivity to bacterial superinfection. PLoS Pathog. 10, e1003885 10.1371/journal.ppat.1003885 24453977 PMC3894209

[43] Deroost K., Pham T.-T., Opdenakker G. and Van den Steen P.E. (2016) The immunological balance between host and parasite in malaria. FEMS Microbiol. Rev. 40, 208–257 10.1093/femsre/fuv046 26657789

[44] Gowda D.C. and Wu X. (2018) Parasite recognition and signaling mechanisms in innate immune responses to malaria. Front. Immunol. 9, 3006 10.3389/fimmu.2018.03006 30619355 PMC6305727

[45] Kwiatkowski D., Sambou I., Twumasi P., Greenwood B.M., Hill A.V.S., Manogue K.R. et al. (1990) TNF concentration in fatal cerebral, non-fatal cerebral, and uncomplicated Plasmodium falciparum malaria. Lancet North Am. Ed. 336, 1201–1204 10.1016/0140-6736(90)92827-5 1978068

[46] Stuart K.D., Weeks R., Guilbride L. and Myler P.J. (1992) Molecular organization of Leishmania RNA virus 1. Proc. Natl. Acad. Sci. 89, 8596–8600 10.1073/pnas.89.18.8596 1382295 PMC49967

[47] de Carvalho R.V.H., Lima-Junior D.S., da Silva M.V.G., Dilucca M., Rodrigues T.S., Horta C.V. et al. (2019) Leishmania RNA virus exacerbates Leishmaniasis by subverting innate immunity via TLR3-mediated NLRP3 inflammasome inhibition. Nat. Commun. 10, 5273 10.1038/s41467-019-13356-2 31754185 PMC6872735

[48] Shio M.T., Eisenbarth S.C., Savaria M., Vinet A.F., Bellemare M.J., Harder K.W. et al. (2009) Malarial hemozoin activates the NLRP3 inflammasome through Lyn and Syk kinases. PLoS Pathog. 5, e1000559 10.1371/journal.ppat.1000559 19696895 PMC2722371

[49] Griffith J.W., Sun T., McIntosh M.T. and Bucala R. (2009) Pure Hemozoin is inflammatory in vivo and activates the NALP3 inflammasome via release of uric acid. J. Immunol. (Baltimore, Md: 1950) 183, 5208–5220 10.4049/jimmunol.0713552 19783673 PMC3612522

[50] Velagapudi R., Kosoko A.M. and Olajide O.A. (2019) Induction of neuroinflammation and neurotoxicity by synthetic hemozoin. Cell. Mol. Neurobiol. 39, 1187–1200 10.1007/s10571-019-00713-4 31332667 PMC6764936

[51] Becker I., Salaiza N., Aguirre M., Delgado J., Carrillo-Carrasco N., Kobeh L.G. et al. (2003) Leishmania lipophosphoglycan (LPG) activates NK cells through toll-like receptor-2. Mol. Biochem. Parasitol. 130, 65–74 10.1016/S0166-6851(03)00160-9 12946842

[52] Storm J. and Craig A.G. (2014) Pathogenesis of cerebral malaria–inflammation and cytoadherence. Front. Cell. Infection Microbiol. 4, 100 10.3389/fcimb.2014.00100 PMC4114466 25120958

[53] de Carvalho R.V.H., Lima-Júnior D.S., de Oliveira C.V. and Zamboni D.S. (2021) Endosymbiotic RNA virus inhibits Leishmania-induced caspase-11 activation. iScience 24, 102004 10.1016/j.isci.2020.102004 33490912 PMC7811143

[54] Weiss L.M. and Dubey J.P. (2009) Toxoplasmosis: a history of clinical observations. Int. J. Parasitol. 39, 895–901 10.1016/j.ijpara.2009.02.004 19217908 PMC2704023

[55] Halonen S.K. and Weiss L.M. (2013) Toxoplasmosis. Handbook Clin. Neurol. 114, 125–145 10.1016/B978-0-444-53490-3.00008-X PMC4157368 23829904

[56] Peterson K.M., Shu J., Duggal P., Haque R., Mondal D. and Petri W.A. Jr (2010) Association between TNF-α and Entamoeba histolytica diarrhea. Am. J. Trop. Med. Hyg. 82, 620 10.4269/ajtmh.2010.09-0493 20348510 PMC2844574

[57] Guo X., Stroup S. and Houpt E. (2008) Persistence of Entamoeba histolytica infection in CBA mice owes to intestinal IL-4 production and inhibition of protective IFN-γ. Mucosal Immunol. 1, 139–146 10.1038/mi.2007.18 19079171

[58] Hamano S., Asgharpour A., Stroup S.E., Wynn T.A., Leiter E.H. and Houpt E. (2006) Resistance of C57BL/6 mice to amoebiasis is mediated by nonhemopoietic cells but requires hemopoietic IL-10 production. J. Immunol. 177, 1208–1213 10.4049/jimmunol.177.2.1208 16818779

[59] Dey I. and Chadee K. (2008) Prostaglandin E2 produced by Entamoeba histolytica binds to EP4 receptors and stimulates interleukin-8 production in human colonic cells. Infect. Immun. 76, 5158–5163 10.1128/IAI.00645-08 18710858 PMC2573325

[60] Chou A. and Austin R.L. (2023) Entamoeba histolytica Infection, StatPearls Publishing LLC., StatPearls, StatPearls Publishing Copyright © 2023 32491650

[61] Rafiei A., Ajami A., Hajilooi M. and Etemadi A. (2009) Th-1/Th-2 cytokine pattern in human amoebic colitis. Pakistan J. Biol. Sci.: PJBS 12, 1376–1380 10.3923/pjbs.2009.1376.1380 20128506

[62] Mortimer L., Moreau F., Cornick S. and Chadee K. (2015) The NLRP3 inflammasome is a pathogen sensor for invasive Entamoeba histolytica via activation of α5β1 integrin at the macrophage-amebae intercellular junction. PLoS Pathog. 11, e1004887 10.1371/journal.ppat.1004887 25955828 PMC4425650

[63] Mortimer L., Moreau F., Cornick S. and Chadee K. (2014) Gal-lectin-dependent contact activates the inflammasome by invasive Entamoeba histolytica. Mucosal Immunol. 7, 829–841 10.1038/mi.2013.100 24253103

[64] Dunst J., Kamena F. and Matuschewski K. (2017) Cytokines and chemokines in cerebral malaria pathogenesis. Front. Cell. Infection Microbiol. 7, 324 10.3389/fcimb.2017.00324 PMC5517394 28775960

[65] Strangward P., Haley M.J., Albornoz M.G., Barrington J., Shaw T., Dookie R. et al. (2018) Targeting the IL33-NLRP3 axis improves therapy for experimental cerebral malaria. PNAS 115, 7404–7409 10.1073/pnas.1801737115 29954866 PMC6048513

[66] Pack A.D., Schwartzhoff P.V., Zacharias Z.R., Fernandez-Ruiz D., Heath W.R., Gurung P. et al. (2021) Hemozoin-mediated inflammasome activation limits long-lived anti-malarial immunity. Cell Rep. 36, 109586 10.1016/j.celrep.2021.109586 34433049 PMC8432597

[67] Kalantari P., DeOliveira R.B., Chan J., Corbett Y., Rathinam V., Stutz A. et al. (2014) Dual engagement of the NLRP3 and AIM2 inflammasomes by plasmodium-derived hemozoin and DNA during malaria. Cell Rep. 6, 196–210 10.1016/j.celrep.2013.12.014 24388751 PMC4105362

[68] Yu X., Du Y., Cai C., Cai B., Zhu M., Xing C. et al. (2018) Inflammasome activation negatively regulates MyD88-IRF7 type I IFN signaling and anti-malaria immunity. Nat. Commun. 9, 4964 10.1038/s41467-018-07384-7 30470758 PMC6251914

[69] Marty-Roix R., Vladimer G.I., Pouliot K., Weng D., Buglione-Corbett R., West K. et al. (2016) Identification of QS-21 as an Inflammasome-activating molecular component of saponin adjuvants. J. Biol. Chem. 291, 1123–1136 10.1074/jbc.M115.683011 26555265 PMC4714196

[70] O'neill L.A., Golenbock D. and Bowie A.G. (2013) The history of Toll-like receptors—redefining innate immunity. Nat. Rev. Immunol. 13, 453–460 10.1038/nri3446 23681101

[71] Vera-Izaguirre D.S., Vega-Memije E., Quintanilla-Cedillo M.R. and Arenas R. (2006) Leishmaniasis. A Review 4, 252–260, Dermatología Cosmética, Médica Y Quirúrgica

[72] Gorfu G., Cirelli K.M., Melo M.B., Mayer-Barber K., Crown D., Koller B.H. et al. (2014) Dual role for inflammasome sensors NLRP1 and NLRP3 in murine resistance to Toxoplasma gondii. mBio 5, 10.1128/mBio.01117-13 24549849 PMC3944820

[73] Chappuis F., Sundar S., Hailu A., Ghalib H., Rijal S., Peeling R.W. et al. (2007) Visceral leishmaniasis: what are the needs for diagnosis, treatment and control? Nat. Rev. Microbiol. 5, 873–882 10.1038/nrmicro1748 17938629

[74] Desjeux P. (2004) Leishmaniasis: current situation and new perspectives. Comp. Immunol. Microbiol. Infect. Dis. 27, 305–318 10.1016/j.cimid.2004.03.004 15225981

[75] Zhu L., Qi W., Yang G., Yang Y., Wang Y., Zheng L. et al. (2022) Toxoplasma gondii Rhoptry Protein 7 (ROP7) interacts with NLRP3 and promotes inflammasome hyperactivation in THP-1-derived macrophages. Cells 11, 1630 10.3390/cells11101630 35626667 PMC9139738

[76] Elmahallawy E.K., Alkhaldi A.A.M. and Saleh A.A. (2021) Host immune response against leishmaniasis and parasite persistence strategies: A review and assessment of recent research. Biomed. Pharmacotherapy 139, 111671 10.1016/j.biopha.2021.111671 33957562

[77] Andargie T.E. and Ejara E.D. (2015) Pro-and anti-inflammatory cytokines in visceral leishmaniasis. J. Cell Sci. Therapy 6, 1

[78] Voronov E., Dotan S., Gayvoronsky L., White R.M., Cohen I., Krelin Y. et al. (2010) IL-1-induced inflammation promotes development of leishmaniasis in susceptible BALB/c mice. Int. Immunol. 22, 245–257 10.1093/intimm/dxq006 20181656

[79] Gurung P., Lukens J.R. and Kanneganti T.D. (2015) Mitochondria: diversity in the regulation of the NLRP3 inflammasome. Trends Mol. Med. 21, 193–201 10.1016/j.molmed.2014.11.008 25500014 PMC4352396

[80] Lima-Junior D.S., Costa D.L., Carregaro V., Cunha L.D., Silva A.L., Mineo T.W. et al. (2013) Inflammasome-derived IL-1β production induces nitric oxide–mediated resistance to Leishmania. Nat. Med. 19, 909–915 10.1038/nm.3221 23749230

[81] Moreira R.B., Pirmez C., de Oliveira-Neto M.P., Aguiar L.S., Gonçalves A.J.S., Pereira L.O.R. et al. (2017) AIM2 inflammasome is associated with disease severity in tegumentary leishmaniasis caused by Leishmania (V.) braziliensis. Parasite Immunol. 39, e12435 10.1111/pim.12435 28397969

[82] Charmoy M., Hurrell B.P., Romano A., Lee S.H., Ribeiro-Gomes F., Riteau N. et al. (2016) The Nlrp3 inflammasome, IL-1β, and neutrophil recruitment are required for susceptibility to a nonhealing strain of Leishmania major in C57BL/6 mice. Eur. J. Immunol. 46, 897–911 10.1002/eji.201546015 26689285 PMC4828310

[83] Harrington V., Gurung P. (2020) Reconciling protective and pathogenic roles of the NLRP3 inflammasome in leishmaniasis. Immunol. Rev. 297, 53–66 10.1111/imr.12886 32564424 PMC7643606

[84] Gurung P., Karki R., Vogel P., Watanabe M., Bix M., Lamkanfi M. et al. (2015) An NLRP3 inflammasome-triggered Th2-biased adaptive immune response promotes leishmaniasis. J. Clin. Invest. 125, 1329–1338 10.1172/JCI79526 25689249 PMC4362229

[85] Lima-Junior D.S., Mineo T.W.P., Calich V.L.G. and Zamboni D.S. (2017) Dectin-1 activation during leishmania amazonensis phagocytosis prompts Syk-dependent reactive oxygen species production to trigger inflammasome assembly and restriction of parasite replication. J. Immunol. (Baltimore, Md: 1950) 199, 2055–2068 10.4049/jimmunol.1700258 28784846

[86] Chaves M.M., Sinflorio D.A., Thorstenberg M.L., Martins M.D.A., Moreira-Souza A.C.A., Rangel T.P. et al. (2019) Non-canonical NLRP3 inflammasome activation and IL-1β signaling are necessary to L. amazonensis control mediated by P2X7 receptor and leukotriene B4. PLoS Pathog. 15, e1007887 10.1371/journal.ppat.1007887 31233552 PMC6622556

[87] Gupta G., Santana A.K.M., Gomes C.M., Turatti A., Milanezi C.M., Bueno Filho R. et al. (2019) Inflammasome gene expression is associated with immunopathology in human localized cutaneous leishmaniasis. Cell. Immunol. 341, 103920 10.1016/j.cellimm.2019.04.008 31078283

[88] de Carvalho R.V.H., Andrade W.A., Lima-Junior D.S., Dilucca M., de Oliveira C.V., Wang K. et al. (2019) Leishmania lipophosphoglycan triggers caspase-11 and the non-canonical activation of the NLRP3 inflammasome. Cell Reports 26, 429.e5–437.e5 10.1016/j.celrep.2018.12.047 30625325 PMC8022207

[89] Shio M.T., Christian J.G., Jung J.Y., Chang K.P. and Olivier M. (2015) PKC/ROS-mediated NLRP3 inflammasome activation is attenuated by leishmania zinc-metalloprotease during infection. PLoS Negl. Trop. Dis. 9, e0003868 10.1371/journal.pntd.0003868 26114647 PMC4482689

[90] Gupta A.K., Ghosh K., Palit S., Barua J., Das P.K. and Ukil A. (2017) Leishmania donovani inhibits inflammasome-dependent macrophage activation by exploiting the negative regulatory proteins A20 and UCP2. FASEB J.: Off. Publ. Federation Am. Societies Exp. Biol. 31, 5087–5101 10.1096/fj.201700407R 28765172

[91] Innes E. (2010) A brief history and overview of Toxoplasma gondii. Zoonoses and Public Health 57, 1–7 10.1111/j.1863-2378.2009.01276.x 19744303

[92] Tait E.D. and Hunter C.A. (2009) Advances in understanding immunity to Toxoplasma gondii. Mem. Inst. Oswaldo Cruz. 104, 201–210 10.1590/S0074-02762009000200013 19430645

[93] Braun L., Brenier-Pinchart M.-P., Yogavel M., Curt-Varesano A., Curt-Bertini R.-L., Hussain T. et al. (2013) A Toxoplasma dense granule protein, GRA24, modulates the early immune response to infection by promoting a direct and sustained host p38 MAPK activation. J. Exp. Med. 210, 2071–2086 10.1084/jem.20130103 24043761 PMC3782045

[94] Ahmed N., French T., Rausch S., Kühl A., Hemminger K., Dunay I.R. et al. (2017) Toxoplasma co-infection prevents Th2 differentiation and leads to a helminth-specific Th1 response. Front. Cell. Infection Microbiol. 7, 341 10.3389/fcimb.2017.00341 PMC5524676 28791259

[95] Oksanen A., Aittomäki S., Jankovic D., Ortutay Z., Pulkkinen K., Hämäläinen S. et al. (2014) Proprotein convertase FURIN constrains Th2 differentiation and is critical for host resistance against Toxoplasma gondii. J. Immunol. 193, 5470–5479 10.4049/jimmunol.1401629 25355923 PMC4261955

[96] Zamboni D.S. and Lima‐Junior D.S. (2015) Inflammasomes in host response to protozoan parasites. Immunol. Rev. 265, 156–171 10.1111/imr.12291 25879291

[97] Quan J.-H., Huang R., Wang Z., Huang S., Choi I.-W., Zhou Y. et al. (2018) P2X7 receptor mediates NLRP3-dependent IL-1β secretion and parasite proliferation in Toxoplasma gondii-infected human small intestinal epithelial cells. Parasites Vectors 11, 1 10.1186/s13071-017-2573-y 29291748 PMC5748956

[98] Moreira-Souza A.C.A., Almeida-da-Silva C.L.C., Rangel T.P., Rocha G.D.C., Bellio M., Zamboni D.S. et al. (2017) The P2X7 receptor mediates toxoplasma gondii control in macrophages through canonical NLRP3 inflammasome activation and reactive oxygen species production. Front Immunol. 8, 1257 10.3389/fimmu.2017.01257 29075257 PMC5643413

[99] Gov L., Schneider C.A., Lima T.S., Pandori W. and Lodoen M.B. (2017) NLRP3 and potassium efflux drive rapid IL-1β release from primary human monocytes during Toxoplasma gondii infection. J. Immunol. (Baltimore, Md: 1950) 199, 2855–2864 10.4049/jimmunol.1700245 28904126 PMC5648586

[100] Pandori W.J., Lima T.S., Mallya S., Kao T.H., Gov L. and Lodoen M.B. (2019) Toxoplasma gondii activates a Syk-CARD9-NF-κB signaling axis and gasdermin D-independent release of IL-1β during infection of primary human monocytes. PLoS Pathog. 15, e1007923 10.1371/journal.ppat.1007923 31449558 PMC6730955

[101] Pazoki H., Mohammad Rahimi H., Mirjalali H., Niyyati M., Mosaffa N., Seyed Tabaei S.J. et al. (2021) Soluble total antigen derived from Toxoplasma gondii tachyzoites increased the expression levels of NLRP1, NLRP3, NLRC4, AIM2, and the release of mature form of IL1β, but downregulated the expression of IL1β and IL18 genes in THP-1cell line. Microb. Pathog. 158, 105072 10.1016/j.micpath.2021.105072 34192597

[102] Pazoki H., Mirjalali H., Niyyati M., Seyed Tabaei S.J., Mosaffa N., Shahrokh S. et al. (2023) Toxoplasma gondii profilin induces NLRP3 activation and IL-1β production/secretion in THP-1 cells. Microb. Pathog. 180, 106120 10.1016/j.micpath.2023.106120 37080500

[103] Kim J.S., Mun S.J., Cho E., Kim D., Son W., Jeon H.I. et al. (2020) Toxoplasma gondii GRA9 regulates the activation of NLRP3 inflammasome to exert anti-septic effects in mice. Int. J. Mol. Sci. 21, 10.3390/ijms21228437 PMC7696177 33182702

[104] Bando H., Lee Y., Sakaguchi N., Pradipta A., Ma J.S., Tanaka S. et al. (2018) Inducible nitric oxide synthase is a key host factor for toxoplasma GRA15-dependent disruption of the gamma interferon-induced antiparasitic human response. mBio 9, 10.1128/mBio.01738-18 30301855 PMC6178625

[105] Wang Y., Cirelli K.M., Barros P.D.C., Sangaré L.O., Butty V., Hassan M.A. et al. (2019) Three Toxoplasma gondii dense granule proteins are required for induction of lewis rat macrophage pyroptosis. mBio 10, 10.1128/mBio.02388-18 e02388–18 PMC6325250 30622189

[106] Bergsbaken T., Fink S.L. and Cookson B.T. (2009) Pyroptosis: host cell death and inflammation. Nat. Rev. Microbiol. 7, 99–109 10.1038/nrmicro2070 19148178 PMC2910423

[107] Stanley S.L. (2003) Amoebiasis. Lancet North Am. Ed. 361, 1025–1034 10.1016/S0140-6736(03)12830-9 12660071

[108] World Health Organization (1998) The World Health Report 1998: Life in the 21st Century A Vision for All: report of the Director-General 241 ISBN 9241561890 ISSN 1020-3311 https://iris.who.int/handle/10665/42065

[109] Guillén N. (2023) Pathogenicity and virulence of Entamoeba histolytica, the agent of amoebiasis. Virulence 14, 2158656 10.1080/21505594.2022.2158656 36519347 PMC9815260

[110] Haque R., Huston C.D., Hughes M. and Houpt E. (2003) Petri Jr WA. Amebiasis. N. Engl. J. Med. 348, 1565–1573 10.1056/NEJMra022710 12700377

[111] Carrero J.C., Reyes-López M., Serrano-Luna J., Shibayama M., Unzueta J., León-Sicairos N. et al. (2020) Intestinal amoebiasis: 160 years of its first detection and still remains as a health problem in developing countries. Int. J. Med. Microbiol. 310, 151358 10.1016/j.ijmm.2019.151358 31587966

[112] Uribe-Querol E. and Rosales C. (2020) Immune response to the enteric parasite entamoeba histolytica. Physiology 35, 244–260 10.1152/physiol.00038.2019 32490746

[113] Kantor M., Abrantes A., Estevez A., Schiller A., Torrent J., Gascon J. et al. (2018) Entamoeba histolytica: updates in clinical manifestation, pathogenesis, and vaccine development. Canadian J. Gastroenterol. Hepatol. 2018, 4601420 10.1155/2018/4601420 PMC6304615 30631758

[114] Galván-Moroyoqui J.M., del Carmen Domínguez-Robles M. and Meza I. (2011) Pathogenic bacteria prime the induction of Toll-like receptor signalling in human colonic cells by the Gal/GalNAc lectin Carbohydrate Recognition Domain of Entamoeba histolytica. Int. J. Parasitol. 41, 1101–1112 10.1016/j.ijpara.2011.06.003 21787776

[115] Sharma M., Bhasin D. and Vohra H. (2008) Differential induction of immunoregulatory circuits of phagocytic cells by Gal/Gal NAc lectin from pathogenic and nonpathogenic Entamoeba. J. Clin. Immunol. 28, 542–557 10.1007/s10875-008-9184-5 18551358

[116] Ngobeni R., Abhyankar M.M., Jiang N.M., Farr L.A., Samie A., Haque R. et al. (2017) Entamoeba histolytica–encoded homolog of macrophage migration inhibitory factor contributes to mucosal inflammation during amebic colitis. J. Infect. Dis. 215, 1294–1302 10.1093/infdis/jix076 28186296 PMC5853319

[117] St-Pierre J., Moreau F., Cornick S., Quach J., Begum S., Aracely Fernandez L. et al. (2017) The macrophage cytoskeleton acts as a contact sensor upon interaction with Entamoeba histolytica to trigger IL-1β secretion. PLoS Pathog. 13, e1006592 10.1371/journal.ppat.1006592 28837696 PMC5587335

[118] Quach J., Moreau F., Sandall C. and Chadee K. (2019) Entamoeba histolytica-induced IL-1β secretion is dependent on caspase-4 and gasdermin D. Mucosal Immunol. 12, 323–339 10.1038/s41385-018-0101-9 30361535

[119] Maldonado‐Bernal C., Kirschning C., Rosenstein Y., Rocha L., Rios‐Sarabia N., Espinosa‐Cantellano M. et al. (2005) The innate immune response to Entamoeba histolytica lipopeptidophosphoglycan is mediated by toll‐like receptors 2 and 4. Parasite Immunol. 27, 127–137 10.1111/j.1365-3024.2005.00754.x 15910421

[120] Haque R., Mondal D., Shu J., Roy S., Kabir M., Davis A.N. et al. (2007) Correlation of interferon-gamma production by peripheral blood mononuclear cells with childhood malnutrition and susceptibility to amebiasis. Am. J. Trop. Med. Hyg. 76, 340–344 10.4269/ajtmh.2007.76.340 17297046

[121] Houpt E.R., Glembocki D.J., Obrig T.G., Moskaluk C.A., Lockhart L.A., Wright R.L. et al. (2002) The mouse model of amebic colitis reveals mouse strain susceptibility to infection and exacerbation of disease by CD4+ T cells. J. Immunol. 169, 4496–4503 10.4049/jimmunol.169.8.4496 12370386

[122] Guo X., Barroso L., Lyerly D.M., Petri W.A. Jr and Houpt E.R. (2011) CD4+ and CD8+ T cell-and IL-17-mediated protection against Entamoeba histolytica induced by a recombinant vaccine. Vaccine 29, 772–777 10.1016/j.vaccine.2010.11.013 21095257 PMC3014458

[123] Rojas-López A., Soldevila G., Meza-Pérez S., Dupont G., Ostoa-Saloma P., Wurbel M. et al. (2012) CCR9+ T cells contribute to the resolution of the inflammatory response in a mouse model of intestinal amoebiasis. Immunobiology. 217, 795–807 10.1016/j.imbio.2012.04.005 22633147

[124] Begum S., Gorman H., Chadha A. and Chadee K. (2020) Role of inflammasomes in innate host defense against Entamoeba histolytica. J. Leucocyte Biol. 108, 801–812 10.1002/JLB.3MR0420-465R 32498132

[125] Martinon F., Mayor A. and Tschopp J. (2009) The inflammasomes: guardians of the body. Annu. Rev. Immunol. 27, 229–265 10.1146/annurev.immunol.021908.132715 19302040

[126] Conos S.A., Lawlor K., Vaux D., Vince J.E. and Lindqvist L. (2016) Cell death is not essential for caspase-1-mediated interleukin-1β activation and secretion. Cell Death Differentiation 23, 1827–1838 10.1038/cdd.2016.69 27419363 PMC5071572

[127] Marie C., Verkerke H.P., Theodorescu D. and Petri W.A. (2015) A whole-genome RNAi screen uncovers a novel role for human potassium channels in cell killing by the parasite Entamoeba histolytica. Sci. Rep. 5, 13613 10.1038/srep13613 26346926 PMC4561901

[128] Li X., Feng M., Zhao Y., Zhang Y., Zhou R., Zhou H. et al. (2021) A novel TLR4-binding domain of peroxiredoxin from Entamoeba histolytica triggers NLRP3 inflammasome activation in macrophages. Front Immunol. 12, 758451 10.3389/fimmu.2021.758451 34659265 PMC8515043

[129] Tasken K. and Aandahl E.M. (2004) Localized effects of cAMP mediated by distinct routes of protein kinase A. Physiol. Rev. 84, 137–167 10.1152/physrev.00021.2003 14715913

[130] Duncan J.A., Bergstralh D.T., Wang Y., Willingham S.B., Ye Z., Zimmermann A.G. et al. (2007) Cryopyrin/NALP3 binds ATP/dATP, is an ATPase, and requires ATP binding to mediate inflammatory signaling. Proc. Natl. Acad. Sci. 104, 8041–8046 10.1073/pnas.0611496104 17483456 PMC1876568

[131] El-Sayed N.M., Myler P.J., Bartholomeu D.C., Nilsson D., Aggarwal G., Tran A.-N. et al. (2005) The genome sequence of Trypanosoma cruzi, etiologic agent of Chagas disease. Science 309, 409–415 10.1126/science.1112631 16020725

[132] mondiale de la Santé O.and Organization WH (2015) Chagas disease in Latin America: an epidemiological update based on 2010 estimates. Weekly Epidemiological Record = Relevé épidémiologique Hebdomadaire 90, 33–44 25671846

[133] Coura J.R. and Viñas P.A. (2010) Chagas disease: a new worldwide challenge. Nature 465, S6–S7 10.1038/nature09221 20571554

[134] Anderson R.M. and May R.M. (1992) Infectious diseases of humans: dynamics and control, Oxford university press

[135] Rassi A. Jr, Rassi A. and Marin-Neto J.A. (2010) Chagas disease. Lancet North Am. Ed. 375, 1388–1402 10.1016/S0140-6736(10)60061-X 20399979

[136] Zingales B. (2018) Trypanosoma cruzi genetic diversity: Something new for something known about Chagas disease manifestations, serodiagnosis and drug sensitivity. Acta Trop. 184, 38–52 10.1016/j.actatropica.2017.09.017 28941731

[137] Acquatella H. (2007) Echocardiography in Chagas heart disease. Circulation 115, 1124–1131 10.1161/CIRCULATIONAHA.106.627323 17339570

[138] Acevedo G., Girard M. and Gomez K. (2018) The unsolved jigsaw puzzle of the immune response in Chagas disease. Front Immunol. 9, 1929 10.3389/fimmu.2018.01929 30197647 PMC6117404

[139] Cerbán F.M., Stempin C.C., Volpini X., Silva E.A.C., Gea S., Motran C.C., (2020) Signaling pathways that regulate*Trypanosoma cruzi* infection and immune response. Biochim Biophys Acta Mol Basis Dis. 1866, 5 165707 10.1016/j.bbadis.2020.165707 32004621

[140] Abrahamsohn I.A. and Coffman R.L. (1996) Trypanosoma cruzi:IL-10, TNF, IFN-γ, and IL-12 regulate innate and acquired immunity to infection. Exp. Parasitol. 84, 231–244 10.1006/expr.1996.0109 8932773

[141] Queiroga T.B.D., Pereira N.S., da Silva D.D., Andrade C.M., de Araújo Júnior R.F., Brito C. et al. (2021) Virulence of Trypanosoma cruzi Strains is related to the differential expression of innate immune receptors in the heart. Front. Cell. Infection Microbiol. 11, 696719 10.3389/fcimb.2021.696719 PMC8321543 34336720

[142] Gonçalves V.M., Matteucci K.C., Buzzo C.L., Miollo B.H., Ferrante D., Torrecilhas A.C. et al. (2013) NLRP3 controls Trypanosoma cruzi infection through a caspase-1-dependent IL-1R-independent NO production. PLoS Negl. Trop. Dis. 7, e2469 10.1371/journal.pntd.0002469 24098823 PMC3789781

[143] Pacheco A.L., Vicentini G., Matteucci K.C., Ribeiro R.R., Weinlich R. and Bortoluci K.R. (2019) The impairment in the NLRP3-induced NO secretion renders astrocytes highly permissive to T. cruzi replication. J. Leukoc. Biol. 106, 201–207 10.1002/JLB.4AB1118-416RR 30997938

[144] Silva G.K., Costa R.S., Silveira T.N., Caetano B.C., Horta C.V., Gutierrez F.R. et al. (2013) Apoptosis-associated speck-like protein containing a caspase recruitment domain inflammasomes mediate IL-1β response and host resistance to Trypanosoma cruzi infection. J. Immunol. (Baltimore, Md: 1950) 191, 3373–3383 10.4049/jimmunol.1203293 23966627

[145] Dey N., Sinha M., Gupta S., Gonzalez M.N., Fang R., Endsley J.J. et al. (2014) Caspase-1/ASC inflammasome-mediated activation of IL-1β-ROS-NF-κB pathway for control of Trypanosoma cruzi replication and survival is dispensable in NLRP3-/- macrophages. PloS ONE 9, e111539 10.1371/journal.pone.0111539 25372293 PMC4221042

[146] Huante M.B., Gupta S., Calderon V.C., Koo S.J., Sinha M., Luxon B.A. et al. (2016) Differential inflammasome activation signatures following intracellular infection of human macrophages with Mycobacterium bovis BCG or Trypanosoma cruzi. Tuberculosis (Edinb.) 101s, S35–S44 10.1016/j.tube.2016.09.026 27733245 PMC7418480

[147] Rojas Márquez J.D., Ana Y., Baigorrí R.E., Stempin C.C. and Cerban F.M. (2018) Mammalian target of rapamycin inhibition in Trypanosoma cruzi-infected macrophages leads to an intracellular profile that is detrimental for infection. Front Immunol. 9, 313 10.3389/fimmu.2018.00313 29515594 PMC5826284

[148] Rodriguez T., Pacheco-Fernández T., Vázquez-Mendoza A., Nieto-Yañez O., Juárez-Avelar I., Reyes J.L. et al. (2020) MGL1 receptor plays a key role in the control of T. cruzi infection by increasing macrophage activation through modulation of ERK1/2, c-Jun, NF-κB and NLRP3 pathways. Cells 9, 108 10.3390/cells9010108 31906385 PMC7017267

[149] Paroli A.F., Gonzalez P.V., Díaz-Luján C., Onofrio L.I., Arocena A. and Cano R.C. (2018) Inflammasome and Caspase-1/11 pathway orchestrate different outcomes in the host protection against Trypanosoma cruzi acute infection. Front Immunol. 9, 913, et al. NLRP3 10.3389/fimmu.2018.00913 29774028 PMC5944318

[150] O'Dempsey T. (2010) CHAPTER 64 - Helminthic infections. In Antibiotic and Chemotherapy Ninth Edition(Finch R.G., Greenwood D., Norrby S.R. and Whitley R.J., eds), 842–859, W.B. Saunders, London

[151] Jourdan P.M., Lamberton P.H.L., Fenwick A. and Addiss D.G. (2018) Soil-transmitted helminth infections. Lancet (London, England) 391, 252–265 10.1016/S0140-6736(17)31930-X 28882382

[152] McSorley H.J. and Maizels R.M. (2012) Helminth infections and host immune regulation. Clin. Microbiol. Rev. 25, 585–608 10.1128/CMR.05040-11 23034321 PMC3485755

[153] Elliott D.E., Summers R.W. and Weinstock J.V. (2007) Helminths as governors of immune-mediated inflammation. Int. J. Parasitol. 37, 457–464 10.1016/j.ijpara.2006.12.009 17313951

[154] Allen J.E. and Maizels R.M. (2011) Diversity and dialogue in immunity to helminths. Nat. Rev. Immunol. 11, 375–388 10.1038/nri2992 21610741

[155] Anthony R.M., Rutitzky L.I., Urban J.F., Stadecker M.J. and Gause W.C. (2007) Protective immune mechanisms in helminth infection. Nat. Rev. Immunol. 7, 975–987 10.1038/nri2199 18007680 PMC2258092

[156] Mahanty S., Mollis S.N., Ravichandran M., Abrams J.S., Kumaraswami V., Jayaraman K. et al. (1996) High levels of spontaneous and parasite antigen-driven interieukin-10 production are associated with antigen-specific hyporesponsiveness in human lymphatic filariasis. J. Infect. Dis. 173, 769–772 10.1093/infdis/173.3.769 8627051

[157] Steel C. and Nutman T.B. (2003) CTLA-4 in filarial infections: implications for a role in diminished T cell reactivity. J. Immunol. 170, 1930–1938 10.4049/jimmunol.170.4.1930 12574361

[158] Sartono E., Kruize Y.C., Kurniawan A., Maizels R.M. and Yazdanbakhsh M. (1997) Depression of antigen-specific interleukin-5 and interferon-γ responses in human lymphatic filariasis as a function of clinical status and age. J. Infect. Dis. 175, 1276–1280 10.1086/593701 9129104

[159] Babu S., Bhat S.Q., Pavan Kumar N., Lipira A.B., Kumar S., Karthik C. et al. (2009) Filarial lymphedema is characterized by antigen-specific Th1 and th17 proinflammatory responses and a lack of regulatory T cells. PLoS Negl. Trop. Dis. 3, e420 10.1371/journal.pntd.0000420 19381284 PMC2666805

[160] Ritter M., Gross O., Kays S., Ruland J., Nimmerjahn F., Saijo S. et al. (2010) Schistosoma mansoni triggers Dectin-2, which activates the Nlrp3 inflammasome and alters adaptive immune responses. Proc. Natl. Acad. Sci. 107, 20459–20464 10.1073/pnas.1010337107 21059925 PMC2996650

[161] Celias D.P., Motrán C.C. and Cervi L. (2020) Helminths turning on the NLRP3 inflammasome: pros and cons. Trends Parasitol. 36, 87–90 10.1016/j.pt.2019.10.012 31753545

[162] Ritter M., Gross O., Kays S., Ruland J., Nimmerjahn F., Saijo S. et al. (2010) Schistosoma mansoni triggers Dectin-2, which activates the Nlrp3 inflammasome and alters adaptive immune responses. PNAS 107, 20459–20464 10.1073/pnas.1010337107 21059925 PMC2996650

[163] Meng N., Xia M., Lu Y.Q., Wang M., Boini K.M., Li P.L. et al. (2016) Activation of NLRP3 inflammasomes in mouse hepatic stellate cells during Schistosoma J. infection. Oncotarget 7, 39316–39331 10.18632/oncotarget.10044 27322427 PMC5129935

[164] Yu Y.-R., Ni X.-Q., Huang J., Zhu Y.-H. and Qi Y.-F. (2016) Taurine drinking ameliorates hepatic granuloma and fibrosis in mice infected with Schistosoma japonicum. Int. J. Parasitol.: Drugs Drug Resistance 6, 35–43 10.1016/j.ijpddr.2016.01.003 PMC4805782 27054062

[165] Liu X., Zhang Y.R., Cai C., Ni X.Q., Zhu Q., Ren J.L. et al. (2019) Taurine alleviates schistosoma-induced liver injury by inhibiting the TXNIP/NLRP3 inflammasome signal pathway and pyroptosis. Infect. Immun. 87, e00732–19 10.1128/IAI.00732-19 31570558 PMC6867867

[166] Celias D.P., Corvo I., Silvane L., Tort J.F., Chiapello L.S., Fresno M. et al. (2019) Cathepsin L3 from fasciola hepatica induces NLRP3 inflammasome alternative activation in murine dendritic cells. Front Immunol. 10, 552 10.3389/fimmu.2019.00552 30967874 PMC6438957

[167] Alvarado R., To J., Lund M.E., Pinar A., Mansell A., Robinson M.W. et al. (2017) The immune modulatory peptide FhHDM-1 secreted by the helminth Fasciola hepatica prevents NLRP3 inflammasome activation by inhibiting endolysosomal acidification in macrophages. FASEB J.: Off. Publ. Federation Am. Societies Exp. Biol. 31, 85–95 10.1096/fj.201500093r 27682204

[168] Jin X., Bai X., Yang Y., Ding J., Shi H., Fu B. et al. (2020) NLRP3 played a role in Trichinella spiralis-triggered Th2 and regulatory T cells response. Vet. Res. 51, 107 10.1186/s13567-020-00829-2 32854770 PMC7457311

